# Evaluation of approaches for multiple imputation of three-level data

**DOI:** 10.1186/s12874-020-01079-8

**Published:** 2020-08-12

**Authors:** Rushani Wijesuriya, Margarita Moreno-Betancur, John B. Carlin, Katherine J. Lee

**Affiliations:** 1 Clinical Epidemiology and Biostatistics Unit, Murdoch Children’s Research Institute, Parkville, VIC 3052 Australia; 2 Clinical Epidemiology and Biostatistics Unit, Department of Paediatrics, University of Melbourne, Parkville, VIC 3052 Australia

**Keywords:** FCS, Joint modelling, Multiple imputation, Multilevel multiple imputation, Three-level data, Incomplete multilevel data, Linear mixed model

## Abstract

**Background:**

Three-level data arising from repeated measures on individuals who are clustered within larger units are common in health research studies. Missing data are prominent in such longitudinal studies and multiple imputation (MI) is a popular approach for handling missing data. Extensions of joint modelling and fully conditional specification MI approaches based on multilevel models have been developed for imputing three-level data. Alternatively, it is possible to extend single- and two-level MI methods to impute three-level data using dummy indicators and/or by analysing repeated measures in wide format. However, most implementations, evaluations and applications of these approaches focus on the context of incomplete two-level data. It is currently unclear which approach is preferable for imputing three-level data.

**Methods:**

In this study, we investigated the performance of various MI methods for imputing three-level incomplete data when the target analysis model is a three-level random effects model with a random intercept for each level. The MI methods were evaluated via simulations and illustrated using empirical data, based on a case study from the Childhood to Adolescence Transition Study, a longitudinal cohort collecting repeated measures on students who were clustered within schools. In our simulations we considered a number of different scenarios covering a range of different missing data mechanisms, missing data proportions and strengths of level-2 and level-3 intra-cluster correlations.

**Results:**

We found that all of the approaches considered produced valid inferences about both the regression coefficient corresponding to the exposure of interest and the variance components under the various scenarios within the simulation study. In the case study, all approaches led to similar results.

**Conclusion:**

Researchers may use extensions to the single- and two-level approaches, or the three-level approaches, to adequately handle incomplete three-level data. The two-level MI approaches with dummy indicator extension or the MI approaches based on three-level models will be required in certain circumstances such as when there are longitudinal data measured at irregular time intervals. However, the single- and two-level approaches with the DI extension should be used with caution as the DI approach has been shown to produce biased parameter estimates in certain scenarios.

## Background

Clustered or multilevel data, in which observations on individual units are correlated because they are nested within clusters, are common in epidemiological research [1]. The clustering in the data may arise due to observational units (usually individuals) being nested within naturally occurring groups such as schools (i.e. cluster correlated data) and/or due to study design such as repeated measurements nested within individuals (i.e. longitudinal data). Clustered data have a naturally hierarchical structure where lower level units are nested within higher level units and there can be multiple levels in this data hierarchy [1]; in particular here we focus on three-level data resulting from the clustering of repeated measures on individuals within larger units such as schools [2]. One such example is provided by the Childhood to Adolescence Transition Study (CATS), a longitudinal study of a cohort of young people recruited just before puberty from schools in Victoria, Australia, and followed up at multiple waves with data collected on a range of mental health outcomes [3].

Missing data present challenges in many studies. In studies such as the CATS, which involve multiple waves of data collection, missing data is a major problem. Multiple imputation (MI), as initially proposed by Rubin (1987), is a popular approach for handling missing data [4]. MI is a two stage process [5]. In the imputation stage, missing values are imputed multiple (*m* > 1) times by sampling from their posterior predictive distribution (or an approximation) using an imputation model based on the available data. In the analysis stage, the *m* completed datasets are analysed using the intended analysis model and the resulting inferences are combined using Rubin’s rules [5]. A key consideration in MI is that in order to generate valid inferences in the substantive analysis, the imputation model needs to preserve all the features of the analysis model such as non-linear relationships, interactions and multilevel features [6]. This has been referred to in the MI literature as congeniality and more recently as substantive-model-compatibility, which is closely related to the concept of congeniality - see Meng (1994) and Bartlett (2015) for formal definitions of these two concepts [7, 8]. In this paper we use the term congeniality, which in the context of a multilevel analysis means that the multilevel structure of the data that will be modelled in the analysis model is accounted for in the imputation model [9, 10]. In the context of an analysis model that is a linear mixed model (LMM), ignoring the multilevel structure during the imputation stage may lead to biased estimates of the regression coefficients and their standard errors, especially when the missing data proportion is large, and also may severely bias estimates of the variance components [9, 11–13].

There are two broad model-based frameworks for imputing missing data in multiple variables; joint modelling (JM) and fully conditional specification (FCS). The JM approach imputes incomplete variables by assuming that they all follow a single joint distribution, for convenience, often a multivariate normal (MVN) distribution [14]. The FCS approach imputes variables with missing values one at a time by using a series of univariate conditional models for each incomplete variable given all the other variables [15, 16]. There is also a variation of the JM approach, which factorizes the joint distribution of the variables into a sequence of conditional distributions [17]. However, we will not consider this approach in the current manuscript due to reasons detailed further in the discussion. The standard JM and FCS methods (referred to as single-level JM and single-level FCS) assume that observations are independent. As a result, when the substantive analysis of interest is a multilevel analysis, imputing using either of these approaches will not be congenial with the analysis model [9, 18]. A simple way of extending the single-level imputation approaches for imputing incomplete two-level data is to include a series of dummy indicators (DIs) to represent the clusters. In addition, from a practical perspective this approach is only sensible when the number of clusters to be represented using DIs is not very large, as a large number of clusters would require a large number of DIs [10], For longitudinal repeated measures data, with follow-ups at fixed intervals of time, an alternative is to arrange the repeated measures of the same variable in wide format and treat each repeated measurement as a distinct variable in the imputation model [19].

Recently, methodologists have extended the JM and FCS approaches to use multilevel imputation models [13, 20–22]. The extension of the JM approach for imputing multilevel data uses a multivariate linear mixed model (MLMM) as the imputation model [20]. Similarly, the multilevel extension to the FCS approach imputes missing values using a series of univariate LMMs [13]. Implementations of both these extensions are now available in a variety of software [23–29], but the majority of these, as well as existing applications and evaluations, are limited to incomplete two-level data [12, 30, 31]. To our knowledge, only the FCS MI approach based on multilevel models has been specifically extended to impute three-level data, and can be implemented in Blimp, which is a stand-alone package for conducting imputation, and the ml.lmer function in the R package ‘miceadds’ [26, 32, 33].

To impute incomplete three-level data researchers may use MI approaches based on three-level imputation models or alternatively, extend single-level or two-level MI approaches by using DIs and/or by analysing repeated measures in wide format for one or both of the levels of clustering. Meanwhile, while the DI and/or analysing repeated measures in wide format do take the three-level structure of the data into account, it is not clear how and when they would produce valid results. No study to date has compared the performance of all these MI approaches in a setting where there are three levels of hierarchy. In fact, methodologists have pointed out that experience in imputing three level data is lacking [34]. Therefore in this paper, we investigate the performance of MI methods for imputing incomplete three-level data, focusing on those that can be used within the mainstream packages R and Stata. For the JM approaches we focus on those assuming a MVN distribution, as this is the most common JM fitted in most statistical packages [35, 36]. Our focus is on multilevel data resulting from repeated measures with follow-ups at fixed intervals of time within an individual where there is clustering among individuals as in the CATS. The analysis model is a LMM with a random intercept for each level of clustering.

The organization of the paper is as follows. We begin with a brief description of the case study and the research question that motivated our study, which aimed to estimate the effect of early depressive symptoms on academic performance. This is followed by a description of the MI approaches we have identified for imputing incomplete three-level data resulting from longitudinal repeated measures across individuals clustered within larger clusters. We then describe a simulation study conducted to evaluate and compare the performance of the approaches, using the CATS example as basis for generating data. An illustration of the various approaches applied to the CATS case study is provided. We conclude with a general discussion.

## Methods

### Motivating case study: the childhood to adolescence transition study (CATS)

The CATS is a longitudinal population-based cohort study conducted in Melbourne, Australia. It is a multidisciplinary study with the long-term goal of studying educational, emotional, social and behavioural development in children from puberty through adolescence [3]. The study recruited Grade 3 students of 8–9 years of age in 2012 from 43 schools. Of the 2289 students enrolled at these schools, 1239 (54%) children with informed parental/guardian consent were recruited into the study at wave 1. Data collection was conducted annually from parent, teacher and student self-report questionnaires along with direct measurements (saliva samples and anthropometric measurements). There is also linkage with the Victorian Curriculum and Assessment Authority (VCAA) to obtain National Assessment Programme – Literacy and Numeracy (NAPLAN) results. NAPLAN, which is administered to all students in schools across Australia in grades 3, 5, 7 and 9 (approximate ages 8–9, 10–11, 12–13, 14–15 years), assess the student’s academic performance on 4 domains – reading, writing, numeracy and language conventions. At the time of this work, we had access to 7 waves of data collection. The detailed study protocol can be found elsewhere [3].

### Target analysis

We focus on an analysis of the effect of early depressive symptoms (at waves 2, 4 and 6) on academic outcome (at waves 3, 5 and 7) as measured by NAPLAN numeracy scores. To account for clustering of individuals within schools and repeated measures within individuals [37], the analysis model used to answer this research question was a LMM for the repeated NAPLAN outcome measures (at the 3 time points) including random effects for school- and child. In this paper we restrict our attention to a random intercept model for brevity, although we note that it may be appropriate to consider a random slope for wave in the applied context. We return to this in the discussion. A measure of depressive symptoms at the previous wave was included as a time-varying exposure along with wave as the time variable. The model was adjusted for potential baseline (wave 1), time-fixed confounders: child’s NAPLAN numeracy scores, sex, socio-economic status (SES), and age [37]:

1 $$ NAPLAN\_{z}_{ij k}={\beta}_0+{\beta}_1\ast {depression}_{ij\left(k-1\right)}+{\beta}_2\ast {wave}_{ij k}+{\beta}_3\ast NAPLAN\_{z}_{ij1}+{\beta}_4\ast {sex}_{ij}+\sum \limits_{a=1}^4\ {\beta}_{5,a}\ast \mathrm{I}\ \left[{SES}_{ij1}=a\right]+{\beta}_6\ast {age}_{ij1}+{\upalpha}_{0i}+{\upalpha}_{0 ij}+{\varepsilon}_{ij k} $$

where *i* denotes the *i*^*th*^ school (*i* = 1, …, 43), *j* denotes the *j*^*th*^ individual (*j* = 1, …1239) and *k* denotes the *k*^*th*^ wave (*k* = 3, 5, 7), with *ε*_*ijk*_ denoting independent random measurement errors distributed as ${\varepsilon}_{ijk}\sim N\ \left(0,{\sigma}_1^2\right)$ and school and individual-level random effects ${\upalpha}_{0i}\sim N\left(0,{\sigma}_3^2\right)$ and ${\upalpha}_{0 ij}\sim N\Big(0,{\sigma}_2^2$) respectively. The I[.] in the above model denotes an indicator function for SES being equal to a. The rest of the notation is described in Table 1.

**Table 1:** Description of the variables measured for the *j*^*th*^ individual belonging to the *i*^*th*^ school at wave *k* in the analysis model

Variable	Type	Grouping /Range	Label
**Child’s sex**	Categorical	0 = Female 1 = Male	*sex*_*ij*_
**Child’s age (wave 1)**	Continuous	Range [7–11]	*age*_*ij*1_
**SES measured by the SEIFA IRSAD quintile (wave 1)**	Categorical	0 = 1st quintile (most disadvantaged)	*SES*_*ij*1_
		1 = 2nd quintile	
		2 = 3rd quintile	
		3 = 4th quintile	
		4 = 5th quintile (most advantaged)	
**Standardized NAPLAN numeracy score (wave 1)**	Continuous	z-score	*NAPLAN* _ *z*_*ij*1_
**Standardized NAPLAN numeracy score (waves 3,5 and 7)**	Continuous	z-score	*NAPLAN* _ *z*_*ijk*_
^a^ **Depressive symptoms (waves 2,4 and 6)**	Continuous	Range [0,8]	*depression*_*ij*(*k* − 1)_
^b^ **Overall child behaviour reported by SDQ (waves 2,4 and 6)**	Continuous	Range[0,40]	*SDQ*_*ij*(*k* − 1)_

*IRSAD* Index of Relative Socio-Economic Advantage and Disadvantage, *NAPLAN* National Assessment Program - Literacy and Numeracy, *SDQ* Strengths and Difficulties Questionnaire, *SEIFA* Socioeconomic Index for Areas, *SES* Socio-Economic Status

^a^A subset of 4 items (each ranging from 0 to 2) from the Short Mood and Feelings Questionnaire (SMFQ) was used to measure the depressive symptoms at each wave in the CATS study [3, 38]. Depressive symptoms at each wave in our study is the total summary score of these four items

^b^ For measuring the overall child behaviour, a total difficulties score is derived from the first 4 subscales of the Strengths and Difficulties Questionnaire (SDQ): emotional symptoms, conduct problems, hyperactivity/inattention, peer relationship problems (each ranging from 0 to 10) [39]. This variable is not included in the analysis but is included in the imputation model as an auxiliary variable to improve its performance

The main target parameter of interest in the above model is *β*_1_, the mean change in standardized NAPLAN numeracy score per unit increase in the depressive symptom score.

In the CATS, all demographic variables in the analysis model, i.e. child’s age, sex and SES quintile, were completely observed [3]. Meanwhile, data were missing for both time-varying variables, that is, NAPLAN numeracy scores and the depressive symptom scores. NAPLAN numeracy scores were missing for 15% (184/1239) of individuals at wave 1, 16% (198/1239) at wave 3, 21% (264/1239) at wave 5, and 30% (366/1239) at wave 7. Depressive symptom scores were missing for 11% (137/1239) of individuals at wave 2, 14% (173/1239) at wave 4 and 21% (249/1239) at wave 6.

### MI methods for handling incomplete three-level data

In the context of repeated measures clustered within larger higher-level clusters such as schools, there are two sources of correlation: the correlation among individuals belonging to the same higher-level cluster and the correlation among the repeated measures of an individual. When these two sources of correlation are accounted for in the analysis model, they need to be accounted for in the imputation model [40]. In this section we outline the MI approaches we have identified for handling incomplete three-level data of this sort, describing how each approach handles these two sources of correlation. We specifically focus on a clustering scenario similar to the CATS where we have a moderate number of higher level clusters and repeated measures at regular intervals of time.

#### Single-level JM with DI for higher level clusters with repeated measures imputed in wide format (JM-1L-DI-wide)

Popularized by Schafer (1997), the single-level JM approach assumes a joint multivariate normal distribution for the incomplete variables. Under this approach imputations for the missing values are drawn from the posterior predictive distribution of the missing data given the observed data using an iterative data augmentation algorithm [14]. With this approach, the within-cluster correlation of the higher-level clusters can be incorporated using a set of dummy variables representing these clusters in the imputation model. Specifically, if there are *I* clusters, the cluster membership of each individual is represented in the imputation model by (*I* − 1) DIs. The clustering of repeated measures within individuals are then imputed in wide format (with one row per individual and separate variables for each repeated measure), and treating them as distinct variables in the imputation model. This approach preserves the clustering of repeated measures in the imputation model by allowing for the correlation between repeated measures with an unstructured covariance matrix.

#### Single level FCS with DI for higher level clusters with repeated measures imputed in wide format (FCS-1L-DI-wide)

The single-level FCS approach specifies a (single-level) univariate imputation model for each incomplete variable. The imputations for the missing values in each variable in turn will be drawn using an iterative algorithm which will cycle through univariate imputation models [16]. Similar to ***JM-1L-DI-wide***, the correlation among individuals belonging to the same higher-level cluster can be modelled through DIs while the correlation among the repeated measures can be modelled by including the repeated measures as distinct variables in the imputation model. Thus, when imputing an incomplete repeated measure at one time point/wave, repeated measures at all the other waves are included as predictors preserving the correlation of the repeated measures.

#### Two-level JM for higher level clusters with repeated measures imputed in wide format (JM-2L-wide)

Schafer and Yucel (2002) extended the JM approach to enable imputation of multilevel data by imputing from a joint MLMM [20]. This multivariate model models the correlation among individuals within a higher-level cluster using cluster-specific random effects which are assumed to follow a normal distribution. As with ***JM-1L-DI-wide***, the clustering of repeated measures within individuals can then be modelled by imputing the data in wide format where repeated measures are treated as distinct variables. In this approach, the incomplete variables are included as outcomes and the complete variables are included as predictors in the imputation model.

#### Two-level FCS for higher level clusters with repeated measures imputed in wide format (FCS-2L-wide)

Van Buuren (2011) proposed an FCS extension for imputing two-level data which uses a series of univariate two-level LMMs to impute the missing values, cycling through the incomplete variables one at a time [13]. Under this approach, repeated measures are treated as distinct variables (imputing the data in wide format) and a univariate two-level LMM is specified for each incomplete repeated measure in turn with cluster-specific random effects to account for the correlation among individuals of the same higher-level cluster.

#### Two-level JM for repeated measures with DI for higher level clusters (JM-2L-DI)

An alternative approach using the two-level MLMM [20], is to use the MLMM to allow for the clustering of repeated measures within an individual, and then model the correlation among individuals of the same higher-level cluster by including DIs representing the cluster membership, imputing the data in long format (i.e. where each repeated measure is a separate row in the dataset).

#### Two-level FCS for repeated measures with DI for higher level clusters (FCS-2L-DI)

Similar to ***JM-2L-DI,*** the two-level FCS approach [13] can be used to model the clustering of repeated measures within individuals using individual specific random effects, with the correlation among individuals of the same higher-cluster modelled using DIs representing the cluster membership, again imputing the data in long format.

#### Three-level JM (JM-3L)

The JM approach for multilevel data [20], can be extended to three levels using a three-level MLMM where the correlation among individuals within the same higher-level cluster is modelled using cluster-specific random effects while the clustering of repeated measures within individuals is modelled using individual-specific random effects applied to the data in long format.

#### Three-level FCS (FCS-3L)

This approach is an extension of the two-level FCS approach proposed by van Buuren (2011) [13] to impute three-level data using a series of univariate three-level LMMs for each variable with missing values [26, 32, 33]. Similar to ***JM-3L***, here the correlation among individuals within the same higher-level cluster is modelled using cluster-specific random effects while the clustering of repeated measures within individuals is modelled using individual-specific random effects imputing the data in long format.

The Table 2 summarizes the approaches discussed above along with the software each of the approach is available in.

**Table 2:** Summary of the imputation approaches for handling incomplete three-level data

MI approach	Paradigm	Model	Software^a^	How the two sources of clustering are handled	
				Clustering due to higher level clusters	Clustering due to repeated measures
***JM-1L-DI-wide***	JM	Standard (single-level)	SAS [64], SPSS [36], Stata [35], Mplus [24], R [46]	DI	Repeated measures arranged in wide format
***FCS-1L-DI-wide***	FCS	Standard (single-level)	SAS, SPSS, Stata, Mplus, R, Blimp [26]	DI	Repeated measures arranged in wide format
***JM-2L-wide***	JM	Two-level MLMM	SAS [28], Mplus, Realcom-impute [23], Stat-JR [29], R	RE	Repeated measures arranged in wide format
***JM-2L-wide***				DI	RE
***FCS-2L-wide***	FCS	Two-level LMM	Mplus, R, Blimp	RE	Repeated measures arranged in wide format
***FCS-2L-DI***				DI	RE
***JM-3L***	JM	Three-level MLMM	Stat-JR, Mplus	RE	RE
***CS-3L***	FCS	Three-level LMM	R, Blimp	RE	RE

*DI* dummy indicators, *FCS* fully conditional specification, *JM* joint modelling, *LMM* linear mixed model, *MLMM* multivariate linear mixed model, *RE* random effects

^a^R and Blimp are the only freely available, open-source software implementations

### Simulation study

We conducted a simulation study based on the CATS case study to compare the performance of the above approaches. In our simulations we did not consider ***JM-3L***, as to our knowledge there are no available implementations of this method in mainstream software. The data were generated as described below, with 40 school cluster (*i* = 1, …, 40) and a total sample size of 1200 students.

#### Generation of the complete data

First we generated 40 school clusters which we populated in two ways to obtain a sample of 1200 students:

1. In the first scenario we assumed that each school contained a fixed cluster size of 30 students, which is a typical class size observed in school setting [41].
2. In the second scenario each school contained a varying number of students ranging from 8 to 66 students, similar to the CATS [3]. In this scenario, the school cluster sizes (8 ≤ *n*_*i*_ ≤ 66) were assumed to follow a truncated log-normal distribution and cluster size for each school *i* was sampled randomly from this distribution. In order to set the total number of students across the 40 schools to be 1200, the sampled cluster sizes were multiplied by a factor of $1200/\sum \limits_{i=1}^{40}{n}_i$ and rounded to derive a scaled class size. If the total of these scaled class sizes was less than 1200, the deficit was added to the last school cluster, if the total of scaled class size was higher than 1200, the excess was deducted from the last school cluster.

Under each of the two scenarios, the rest of the variables were generated sequentially as described below for individual *j* in cluster *i*. The values of the parameters indexing these distributions were determined by estimating the respective quantity from the CATS data and are given in Additional file 1: Table S1.

i. Child’s age at wave 1 (*age*_*ij*1_) was generated from a uniform distribution, *U*(*a*, *b*).
ii. Child’s sex (*sex*_*ij*_) was generated by randomly assigning *λ*% of students to be female.
iii. Child’s SES quintile at wave 1 (*SES*_*ij*1_) was generated by randomly assigning *θ*_0_, (*θ*_1_ − *θ*_0_), (*θ*_2_ − *θ*_1_), (*θ*_3_ − *θ*_2_) and (1 − *θ*_3_) % of respondents to SES quintiles 1,2,3,4 and 5 respectively.
iv. The standardised NAPLAN scores at wave 1 (*NAPLAN* _ *z*_*ij*1_) were generated from a linear regression model conditional on child’s sex, child’s age at wave 1 and child’s SES quintile:

2 $$ NAPLAN\_{z}_{ij1}={\eta}_0+{\eta}_1\ast \left[{sex}_{ij}=1\right]+{\eta}_2\ast {age}_{ij1}+\sum \limits_{a=1}^4{\eta}_{3,a}\ast \mathrm{I}\ \left[{SES}_{ij1}=a\right]+{\psi}_{ij} $$

where *ψ*_*ij*_ are independently and identically (iid) distributed as as ${\psi}_{ij}\sim N\ \left(0,{\sigma}_{\varphi}^2\right)$ and a = 0 (reference category),1,2,3, or 4 representing the SES quintiles 1–5 respectively.

v. Child’s depression status at waves 2, 4 and 6 (*depression*_*ij*(*k* − 1)_) was generated using a LMM conditional on child’s age at wave 1, child’s sex, NAPLAN scores at wave 1, child’s SES quintile and wave:

3 $$ {depression}_{ij k}={\delta}_0+{\delta}_1\ast {age}_{ij1}+{\delta}_2\ast \left[{sex}_{ij}=1\right]+{\delta}_3\ast NAPLAN\_{z}_{ij1}+\sum \limits_{a=1}^4\ {\delta}_{4,a}\ast \mathrm{I}\ \left[{SES}_{ij1}=a\right]+{\delta}_5\ast {wave}_{ij k}+{u}_{0i}+{u}_{0 ij}+{\varphi}_{ij k} $$

Where *φ*_*ijk*_, *u*_0*ij*_ and *u*_0*i*_ are iid as ${\varphi}_{ijk}\sim N\ \left(0,{\sigma}_{\varphi}^2\right)$, ${u}_{0 ij}\sim N\left(0,{\sigma}_{u_2}^2\right),$ and ${u}_{0i}\sim N\left(0,{\sigma}_{u_3}^2\right)$ respectively.

vi. Child’s standardized NAPLAN score at waves 3, 5 and 7 (*NAPLAN* _ *z*_*ijk*_) was generated from a LMM as shown below:

4 $$ NAPLAN\_{z}_{ij k}={\beta}_0+{\beta}_1\ast {depression}_{ij\left(k-1\right)}+{\beta}_2\ast {wave}_{ij k}+{\beta}_3\ast {age}_{ij1}+{\beta}_4\ast \left[{sex}_{ij}=1\right]+{\beta}_5\ast NAPLAN\_{z}_{ij1}+\sum \limits_{a=1}^4{\beta}_{6,a}\ast \mathrm{I}\ \left[{SES}_{ij1}=a\right]+{\upalpha}_{0i}+{\upalpha}_{0 ij}+{\varepsilon}_{ij k} $$

where *ε*_*ijk*_, α_0*i*_ and α_0*ij*_ are iid as $$ {\varepsilon}_{ijk}\sim N\ \left(0,{\sigma}_1^2\right) $$$$ {\upalpha}_{0i}\sim N\left(0,{\sigma}_3^2\right), $$ and $$ {\upalpha}_{0 ij}\sim N\Big(0,{\sigma}_2^2 $$) respectively.

vii. Finally, child’s behavioural problems at waves 2, 4 and 6 (*SDQ*_*ijk*_), which is not included in the analysis but will be included in the imputation model as an auxiliary variable to improve its performance [42], was generated using a LMM conditional on depression symptoms at waves 2, 4 and 6 and wave:

5 $$ {SDQ}_{ijk}={\gamma}_0+{\gamma}_1\ast {depression}_{ijk}++{\gamma}_2\ast {wave}_{ijk}+{\nu}_{0i}+{\nu}_{0 ij}+{\epsilon}_{ijk} $$

where *ϵ*_*ijk*_, *ν*_0*i*_ and *ν*_0*ij*_ are iid as; $$ {\epsilon}_{ijk}\sim N\ \left(0,{\sigma}_{\epsilon}^2\right) $$$$ {\nu}_{0i}\sim N\left(0,{\sigma}_{v_3}^2\right), $$ and $$ {\nu}_{0 ij}\sim N\Big(0,{\sigma}_{v_2}^2 $$) respectively.

Steps i-vii above were replicated 1000 times. This number was selected to limit the Monte Carlo standard error related to the coverage, which with 1000 replications will be approximately 0.7% [43].

In order to compare the performance of the MI approaches under different degrees of correlation at the two levels, we considered two different intra-cluster correlation (ICC) values at level 3 (high = 0.15 and low = 0.05) and at level 2 (high = 0.5 and low = 0.2). This resulted in four simulation scenarios corresponding to four pairs of ICC values: High-High, High-Low, Low-High and Low-Low. These ICC values were chosen based on the estimated ICC values in the CATS (level-2 ICC = 0.5 and level-3 ICC = 0.07) and the literature (an ICC of 0.05 is common in cluster randomized trials [44] and larger ICC values such as 0.2 are seen in repeated measures designs [6, 45]). Under each of the ICC combinations, the respective variance components at levels 1, 2 and 3 in the final population data generating model (Eq. 4) were obtained by equating the total variance across all the three levels to unity (i.e. ${\sigma}_1^2+{\sigma}_2^2+{\sigma}_3^2=1\Big)$ (see Additional file 1: Table S2).

#### Generation of missing data

We set data to missing in depressive symptom scores at waves 2, 4 and 6 (the exposure of interest). Specifically, to mimic the missing data proportions observed in the CATS, 10, 15 and 20% of the depression symptom scores at waves 2, 4 and 6, respectively, were set to missing. To evaluate the approaches under a more extreme example, in a second scenario we set 20, 30 and 40% of the depressive symptom measures at waves 2, 4 and 6 to missing.

We set depressive symptoms to be missing completely at random (MCAR) and according to two missing at random (MAR) mechanisms: MAR-CATS and MAR-inflated. Under the MCAR mechanism, the desired proportion of depression symptom scores at each wave was set to be missing using simple random sampling. Under the MAR mechanisms, depression symptom scores at each wave were set to be missing according to a logistic regression model dependent on the standardized NAPLAN scores at the subsequent wave and the SDQ measure at the concurrent wave:

6 $$ \mathrm{logit}\left(P\left(R\_{depression}_{ij k}=1\right)\right)={\zeta}_{0k}+{\zeta}_1\ast NAPLAN\_{z}_{ij\left(k+1\right)}+{\zeta}_2\ast {SDQ}_{ij k} $$

where, *R* _ *depression*_*ijk*_ is an indicator variable which takes the value 0 if *depression*_*ijk*_ is missing and 1 if *depression*_*ijk*_ is observed.

For the MAR-CATS scenario we used the associations between the probability of response and the predictors of response observed in the CATS. For the MAR-inflated scenario we doubled the values of *ζ*_1_ and *ζ*_2_. The values of the intercepts *ζ*_0k_ were chosen by iteration so that the required proportions of missingness were achieved for each of the waves (2, 4 and 6). The values of the parameters *ζ*_1_ and *ζ*_2_, and the corresponding odds ratios for each of the two MAR scenarios are shown in the Additional file 1: Table S3.

### MI methods and evaluation

For the 24 scenarios considered (2 cluster sizes × 4 ICC combinations × 3 missingness mechanisms), we applied the following 8 MI approaches: ***JM-1L-DI-wide***, ***FCS-1L-DI-wide***, ***JM-2L-wide***, ***FCS-2L-wide, JM-2L-DI, FCS-2L-DI, FCS-3L*** in Blimp and ***FCS-3L*** in ml.lmer to impute missing values in depressive symptom scores at waves 2, 4 and 6 in each of the simulated data sets.

Except for ***FCS-3L*** in Blimp, all the other approaches were implemented in R version 3.5.0 [46]. For the ***FCS-3L*** in Blimp we used the Blimp beta version 1.1 [26]. For the JM approaches, ***JM-1L-DI-wde***, ***JM-2L-wide***, ***JM-2L-DI***, the R package ‘jomo’ was used while for the FCS approaches, ***FCS-1L-DI-wide***, ***FCS-2L-wide, FCS-2L-DI***, the R package ‘mice’ was used. Specifically for the single-level JM approach, the function jomo1con in the ‘jomo’ package was used while for the single-level FCS approach, the function norm in the ‘mice’ package was used. For the two-level JM approaches the function jomo1rancon in the ‘jomo’ package was used and for the two-level FCS approaches the function 2 l.pan in the ‘mice’ package was used. While there are several alternative functions in R for specifying a two-level FCS imputation model, we chose the above because they have been shown to perform well for handling incomplete longitudinal data in previous simulation studies [47]. Although the ***FCS-3L*** implementations in Blimp and ml.lmer both impute missing values using three-level LMMs as the imputation model, in ***FCS-3L*** in ml.lmer the three-level model is fitted using maximum likelihood, while in Blimp a fully Bayesian approach is used. In addition, the ***FCS-3L*** implementation in ml.lmer can also handle non-hierarchical cross-classified data. For regression coefficients and the variance parameters in the imputation model for ***FCS-3L*** in Blimp, we used the default priors specified in Blimp [26].

In addition to all the variables in the analysis model, the imputation model for each of the 8 MI approaches also included child behaviour problems (reported by SDQ) at waves 2, 4 and 6 as auxiliary variables. For each simulated dataset, 20 imputations were generated for each of the MI approaches [5]. After examining trace plots, the JM MI approaches were run after a burn-in of 1000 iterations with 100 between-imputation iterations, and the FCS MI approaches in R a burn-in of 10 iterations. The FCS approach in Blimp were run after a burn-in of 1000 iterations with 100 thinning iterations, as with 1000 iterations the potential scale reduction (PSR) factor values were less than 1.10 which according to the Blimp user guide (version 1.1) is generally acceptable [30].

The target analysis model (Eq. 1) was then fitted to each of the imputed data sets and the estimates were pooled across the imputed datasets using Rubin’s rules [5, 48]. The parameters of interest were the regression coefficient for depressive symptoms (*β*_1_), and the estimates of the variance components at levels 1, 2 and 3 (${\sigma}_1^2,{\sigma}_2^2,{\sigma}_3^2$ respectively). We also conducted an available case analysis (ACA) where waves of an individual with missing values were excluded from the analysis.

The estimates of ${\beta}_1,{\sigma}_1^2,{\sigma}_2^2$ and ${\sigma}_3^2$ from the approaches were compared to the true values of these parameters that were used to simulate the data. In order to compare the performance of the various approaches for estimating the regression coefficient of interest we calculated the bias, the average difference between the true value and the estimates across 1000 replications; the empirical standard error, the average standard deviation of the estimates from 1000 replications; the model-based standard error, the average of the standard error of the estimates across 1000 replications; and the coverage probability, estimated by the proportion of replications where the estimated 95% confidence interval contained the true value [49]. For the variance component estimates, we report the bias and empirical standard error. We also report the percentage bias which is defined as the bias relative to the true value as a percentage.

## Results

### Simulation study

The comparative performance of the MI approaches were very similar for the MCAR, MAR-CATS and MAR-inflated scenarios so we focus on the results from the MAR-CATS scenario.

**Fig. 1:**
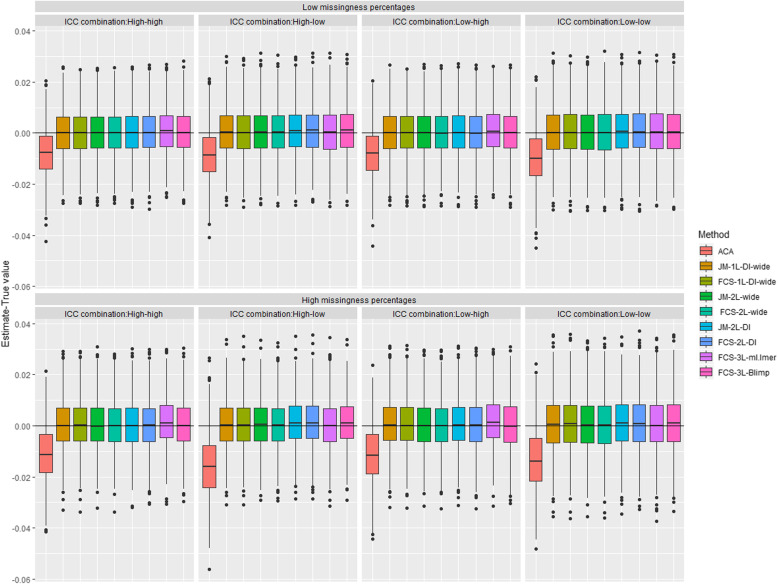
Distribution of the bias in the estimated regression coefficient of interest (*β*_1_, *true value* =  − 0.025) across the 1000 simulated datasets for available case analysis (ACA) and the 8 multiple imputation (MI) approaches under two scenarios for missing data proportions at waves 2, 4 and 6 (10%, 15%, 20% and 20%, 30%, 40%, respectively) and four ICC combinations when data are missing at random (MAR-CATS). The lower and upper margins of the boxes represent the 25th (Q1) and the 75th (Q3) percentiles of the distribution respectively. The whiskers extend to Q1–1.5*(Q3- Q1) at the bottom and Q3 + 1.5*(Q3- Q1) at the top. The following abbreviations are used to denote different MI methods, e.g., DI: dummy indicators, FCS: fully conditional specification, JM: joint modelling

**Fig. 2:**
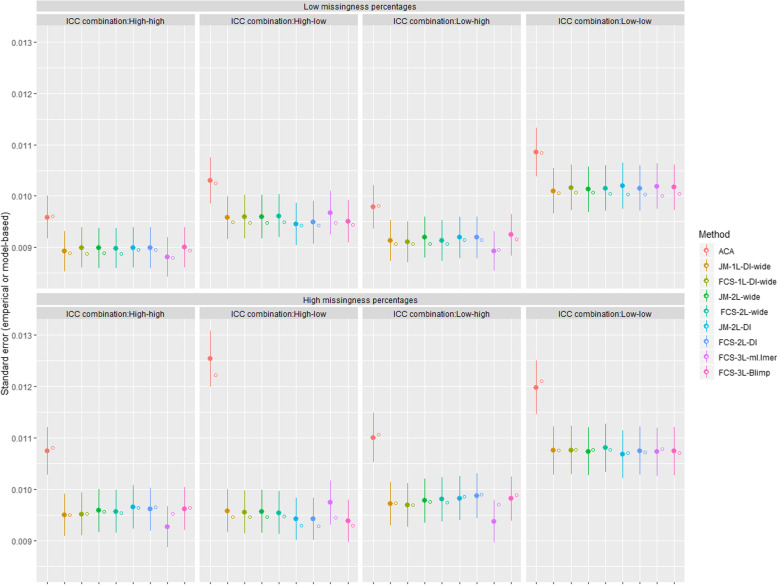
Empirical standard errors (filled circles with error bars showing ±1.96× Monte Carlo standard errors) and average model-based standard errors (hollow circles) from 1000 simulated datasets, for available case analysis (ACA) and the 8 multiple imputation (MI) approaches under two scenarios for missing data proportions at waves 2,4 and 6 (10%, 15%, 20% and 20%, 30%, 40%, respectively) and four ICC combinations when data are missing at random (MAR-CATS). The following abbreviations are used to denote different MI methods, e.g., DI: dummy indicators, FCS: fully conditional specification, JM: joint modelling

The sampling distributions of the estimated bias of the regression coefficient of interest (*β*_1_) across the 1000 replications for each analysis approach for different scenarios are displayed in Fig. 1. As expected, we observed large bias (> 10% relative bias), slightly larger standard errors than with the MI approaches and severe under-coverage (< 0.90) for ACA across all ICC combinations and missing data proportions, all of which increased with lower ICC values and higher missing data proportions. While all the MI approaches showed minimal biases, there were slightly higher biases for ***FCS-3L*** in ml.lmer when the ICC at level 2 was high and with ***JM-2L-DI, FCS-2L-DI,*** and ***FCS-3L*** in Blimp when the ICC at level 2 was low. These biases were more prominent in the MAR-inflated scenario with higher missing data proportions (Table S6). All the MI approaches resulted in comparable empirical and model-based standard errors with coverage probabilities close to the nominal level (Fig. 2 and Table S5).

**Fig. 3:**
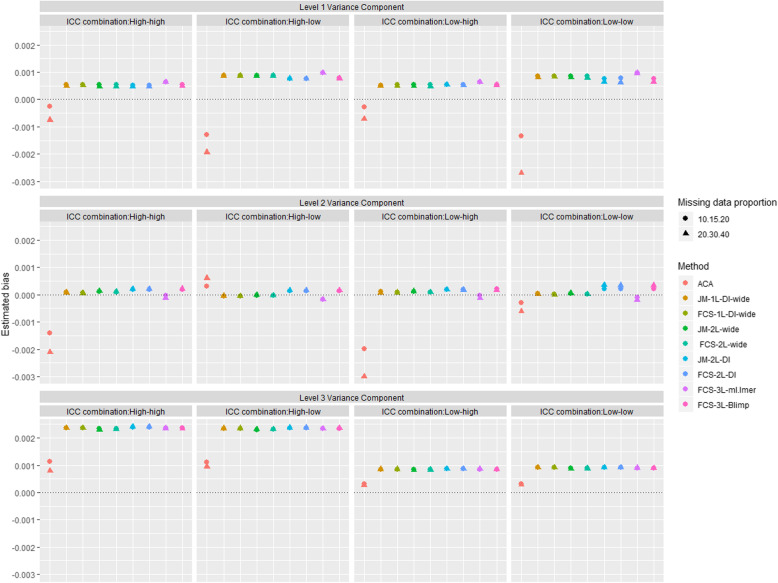
Estimated bias in the variance components at level 1, 2 and 3 across the 1000 simulated datasets available case analysis (ACA) and the 8 multiple imputation (MI) approaches under two scenarios for missing data proportions at waves 2, 4 and 6 (10%, 15%, 20% and 20%, 30%, 40%, respectively) and four ICC combinations when data are missing at random (MAR-CATS). The following abbreviations are used to denote different MI methods, e.g., DI: dummy indicators, FCS: fully conditional specification, JM: joint modelling

Figure 3 shows the estimated biases for the variance components at level 1, 2 and 3 across different simulation scenarios. All approaches resulted in similar negligible bias (< 10% relative bias) for the variance components at level 1, 2 and 3 across the different simulation scenarios. For all MI approaches there were slightly larger biases for the level 3 variance estimates when there was a high ICC at level 3, but level 1 and 2 variance estimates were unbiased across the different ICC combinations and missing data proportions. The ACA approach produced slightly larger bias in the level 2 variance whenever the ICC at level 2 was high, and for the level 1 variance when the ICC at level 2 was low.

The performance of the approaches was similar with variable cluster sizes (results available on request) except when the data were MCAR, where there was slightly higher albeit still minimal bias for ***FCS-2L-DI,FCS-3L*** in ml.lmer and ***FCS-3L*** in blimp for some scenarios with high missing data proportions.

### Application to the CATS data

The MI approaches evaluated in the simulation study were also applied to the CATS data to provide an empirical comparison and these resultes are shown in Table 3. Consistent with the simulation results, the regression coefficient estimates and the standard errors were very similar irrespective of the method of analysis, with slightly more variability in the variance component estimates, especially with ***FCS-3L*** in ml.lmer where we observed a considerably smaller estimate for the level 3 variance component. All the approaches suggest that an increase in the depressive symptom score is associated with a small decrease in the standardized NAPLAN numeracy score at the subsequent wave. Of note it was not possible to apply ***FCS-2L-DI*** for the CATS due to sparse data. The estimated regression coefficients (and standard errors) of the adjusting covariates were also quite similar under the different MI approaches evaluated, and these are shown in the Additional file 1: Table S13.

**Table 3:** Point estimate (and standard error) for the effect of early depressive symptoms on subsequent standardized NAPLAN numeracy scores, and point estimates for the variance components at levels 3, 2 and 1, from available case analysis (ACA) and 8 MI approaches applied to the CATS data analysis

Method	Regression coefficient estimate (SE)	Level 3 variance component	Level 2 variance component	Level 1 variance component
***ACA***	−0.022 (0.007)	0.042	0.239	0.232
***JM-1L-DI-wide***	−0.019 (0.007)	0.043	0.243	0.231
***FCS-1L-DI-wide***	−0.019 (0.008)	0.043	0.246	0.230
***JM-2L-wide***	−0.020 (0.007)	0.041	0.246	0.228
***FCS-2L-wide***	−0.022 (0.007)	0.042	0.245	0.229
***JM-2L-DI***	−0.020 (0.008)	0.042	0.237	0.228
***FCS-2L-DI***	–	–	–	–
***FCS-3L*** in ml.lmer	−0.021 (0.007)	0.033	0.238	0.232
***FCS-3L*** in Blimp	−0.021 (0.007)	0.040	0.238	0.228

*ACA* available case analysis, *DI* dummy indicators, *FCS* fully conditional specification, *JM* joint modelling, *NAPLAN* National Assessment Program - Literacy and Numeracy, *RE* random effects, *SE* standard error

## Discussion

While implementations of several MI approaches for imputing single- and two-level data are available in mainstream statistical software, there are limited options for imputing incomplete three-level data using three-level imputation models. Further, these approaches have not been compared with the pragmatic DI/wide adaptations of the more readily available approaches designed for imputing single- and two-level data presented here. We report a comparison of all these approaches using simulations and a real-data example based on the CATS. All of the MI approaches considered resulted in approximately unbiased estimates for the coefficient of the exposure with confidence intervals achieving nominal coverage across the different ICC combinations and missing data proportions considered in our study. We found similar comparable performance of these approaches in our case study as well. Simulations also showed that, when the cluster sizes varied, ***FCS-2L-DI, FCS-3L*** in ml.lmer, and ***FCS-3L*** in Blimp showed slightly higher biases in the setting with a high proportion of missing data than the other approaches. This suggests that the multilevel FCS approaches could be more sensitive to sparse data resulting from small cluster sizes and missing data than the JM approaches. As expected, severe biases and under-coverage were observed for ACA when data were MAR, with slight gains in precision for all MI approaches compared to ACA [50]. We observed very little bias for the estimates of the variance components for all the approaches, including ACA, under all simulation scenarios. Our finding that the performance of the single-level approaches ***JM-1L-DI-wide***, ***FCS-1L-DI-wide*** was similar to the approaches using three-level imputation models is highly relevant for practice since the commonly used statistical packages Stata and SPSS often used by researchers do not have MI approaches based on multilevel imputation models [18].

The advantage of three-level MI approaches over the pragmatic adaptations of single- and two-level approaches is that they have much lower computational time. In practice as only several imputations are required (generally less than 100) [51], we do not consider this to be a substantial practical limitation. The findings from our study are important as ***FCS-3L*** in ml.lmer is a very recent command that can be challenging to implement due to limited documentation (however, see [52] for a demonstration of its functionality), and ***FCS-3L*** in Blimp is a standalone package. However, the single-level and two-level approaches with DI and repeated measures imputed in wide format may incur convergence issues of the imputation model when there are a large number of clusters and/or a large number of incomplete repeated measures, as with ***FCS-2L-DI*** in our case study. In addition, the methods which impute repeated measures in wide format (***JM-1L-DI-wide***, ***FCS-1L-DI-wide***, ***JM-2L-wide***, and ***FCS-2L-wide)*** for the repeated measures cannot be applied with repeated measures that are not recorded at fixed intervals of time. Previous simulation studies have shown that the DI approach can result in inflated standard errors and biased variance components estimates (and therefore ICCs) particularly when the ICC is low and there are small cluster sizes [12, 18, 40]. Given all of these findings, it is suggested that DI approaches be used with caution.

In the single-level context where all variables are continuous and approximately normally distributed, JM and FCS approaches have been shown to be equivalent [53, 54]. However in the multilevel context, even with normally distributed variables, this is not necessarily true [10]. The multilevel JM approach by Schafer and Yucel (2002), as implemented in R, is slightly different from the standard multilevel FCS implementation in R as originally proposed by van Buuren (2011), because it allows associations between incomplete variables to vary at different levels [10]. The standard multilevel FCS approach can be made equivalent to the multilevel JM approach by including arithmetic means of the imputed variables as cluster means in the imputation model [9]. However, in including the cluster means this way, the FCS approaches assume that the cluster sizes are equal and it is argued that for the FCS approaches to be formally equivalent to the JM approaches in the multilevel context, the cluster sizes should also be equal [9, 31] (Note: the new Blimp version 2.2 also has an additional alternative approach- “latent” approach, for incorporating the group means which do not assume equal cluster sizes) [55]. While these differences between the multilevel JM and FCS could be important in the context of complicated multilevel analyses that assume different associations between variables at different levels, the substantive analysis model considered in the current manuscript does not assume such relations. Therefore as these differences are not largely relevant in the context of our example we do not discuss these differences in more detail. For a detailed discussion of the formal differences between the JM and FCS approaches in the multilevel context, see Carpenter and Kenward (2013),Enders et al. (2016) and Mistler (2017) [9, 10, 56].

No study to date has compared all of the available MI approaches in a three-level data setting. However, our results are consistent with those from similar studies conducted in a two-level setting. In particular, Huque et al. (2018) and Huque et al.(2019) showed that the single-level JM and FCS approaches which impute the repeated measures in wide format to account for the clustering of repeated measures (labelled JM-mvni and FCS-standard in their study) performed well compared to several generalized linear mixed model (GLMM) based approaches for handling incomplete longitudinal data [47, 57]. Our results are also consistent with the simulations result of Enders et al. (2017), who showed that the two-level MI application in Blimp resulted in regression coefficients with negligible bias even in small samples with large proportions of missing data and minimal bias for the variance component estimates for a random intercept model [30]. Several studies that have assessed the DI approach to impute two-level data found that with missing values in predictors, the DI approach produces reliable estimates of the regression coefficients while the estimates of variance components can be biased, especially when the ICC is low coupled with very high missing data rates and small cluster sizes [12, 13, 18, 34, 40]. Consistent with these findings, we observed that with low ICC at level 2, the variance components at level 2 for the ***JM-2L-DI*** and ***FCS-2L-DI*** were slightly more biased than with the other MI approaches.

It is always difficult to draw conclusions from a single simulation study, but the fact that our simulations were based on a real study allowed us to incorporate complex yet realistic associations, meaning that the findings reflect what could be expected in settings with a similar clustering structure. We limited our simulations to a random-intercept analysis model with missing data at level 1 only for brevity. However, caution should be taken when generalizing these results to more complex analysis models, for example multilevel analysis models with random slopes and/or interaction terms. It would be interesting to compare the possible approaches in the context of a random slope model because it is likely that the performance of these approaches are quite different [57]. With random slopes, the single- and two-level imputation models with extensions, particularly those which use DIs, might lead to biased estimates and can often be infeasible with a large number of clusters [58]. In addition, if explanatory variables with random slopes or interaction effects are incomplete, MI as implemented in standard software (the “reversed” imputation strategy) may no longer be valid [31]. Recently introduced substantive-model-compatible (also referred to as model-based) MI approaches could be a potential solution for this problem but is beyond the scope of this paper [8, 59, 60].

An alternative JM approach for imputing multilevel data was described by Asparouhav and Muthén (2010). Similar to the JM approach by Schafer and Yucel (2002), this approach also uses a joint MLMM for imputing incomplete variables but treats all variables, complete and incomplete, as outcomes in the imputation model [61]. This approach is slightly less restrictive than the JM approach by Schafer and Yucel (2002) as it allows associations between all variables to vary at different levels, and as a result can be congenial with more complicated multilevel analysis models that assume different associations between variables (both complete and incomplete) at different levels [7, 56]. We did not include this approach in our study as our substantive analysis model did not assume such associations. Another variation of the JM approach which can be used to impute multi-level data is the sequential parameterization of the joint model. Although more flexible than the commonly implemented JM approach, the specification of separate conditional models for each incomplete variable requires more consideration by the researcher than the JM approach [17]. Also, while there is a recent implementation of this approach that can handle two-level data, we are not aware of any implementations of this approach that can handle three-level data [59]. Although it would be possible to accommodate three-level data using this approach using wide/DI adaptation due to the complexity of this approach this is less appealing than the approaches considered here.

While our study focused on the setting with repeated measures clustered within individuals who are in turn clustered within large groups, the pragmatic methods suggested in our study may be adapted to more general three-level settings. However evaluation of the performance of these adaptations compared to the three-level imputation models in a more general context is still an area for future work. For example, an extension to our study could be to explore the performance of these MI approaches with more complex analysis models or where there are missing data at level 2 (time-fixed variables) and level 3 (cluster-specific variables). In our simulation study missingness was imposed in a continuous variable. Therefore, another extension would be to assess the performance of the MI approaches for imputing mixtures of categorical and continuous variables as the different approaches impute incomplete categorical variables in different ways [62]. Finally, our simulations considered only MCAR and MAR missingness mechanisms and the MI methods evaluated in our simulations are only guaranteed to produce unbiased estimates under MAR. In practice it is possible that the data are missing not at random (MNAR), although this does not preclude unbiased estimation from the approaches considered [63]. Future research should examine the performance of these methods under MNAR mechanisms.

Despite the limitations of this manuscript discussed above, our study is the first to provide a much needed comparison of the currently available approaches for imputing incomplete longitudinal data that are nested within clusters which has important implications for the practical researcher.

## Conclusion

In conclusion, the findings from our study indicate that both single-and two-level MI approaches can be extended with DIs and/or imputing repeated measures in wide format to adequately handle incomplete three-level data, performing as well as the MI approaches using three-level imputation models. Therefore in practice, researchers may choose an appropriate method based on the substantive analysis model, computational time and software preference. However, approaches which use the DI extension should be used with caution as it has been shown to produce biased parameter estimates in certain scenarios. In the presence of longitudinal data measured at irregular time intervals, researchers may have no other choice than the three-level imputation approaches.

## Supplementary information

**Additional file 1: Table S1, S2 and S3.** contains the parameter values used in the data generating and missing data generation models of the simulation study. **Table S4, S5 and S6** contains the values of performance measures, for available case analysis (ACA) and 8 multiple imputation (MI) approaches for estimating the regression coefficient of depressive symptom scores at the previous wave, under the three missing data mechanisms missing completely at random (MCAR), missing at random similar to CATS (MAR-CATS) and inflated missing at random (MAR-inflated) respectively. **Table S7 and S8.** contains the values of performance measures, for ACA and 8 multiple imputation (MI) approaches in estimating the variance components at level 3, 2 and 1, when data are missing completely at random (MCAR) with low (10, 15 and 20% across waves 2, 4 and 6 respectively) and high (20, 30 and 40% across waves 2, 4 and 6 respectively missing data percentages across waves respectively. **Table S9 and S10.** contains the values of performance measures, for ACA and 8 multiple imputation (MI) approaches in estimating the variance components at level 3, 2 and 1, when data missing at random (MAR-CATS) with low (10, 15 and 20% across waves 2, 4 and 6 respectively) and high (20, 30 and 40% across waves 2, 4 and 6 respectively missing data percentages across waves respectively. **Table S11 and S12.** contains the values of performance measures, for ACA and 8 multiple imputation (MI) approaches in estimating the variance components at level 3, 2 and 1, when data are missing at random (MAR-inflated) with low (10, 15 and 20% across waves 2, 4 and 6 respectively) and high (20, 30 and 40% across waves 2, 4 and 6 respectively missing data percentages respectively. **Table S13.** contains the estimated regression coefficients (and standard errors) for the adjusting covariates, from available case analysis (ACA) and 8 MI approaches applied to the CATS data analysis. **Fig. S1 and S2.** shows the distribution of the bias in the estimated regression coefficient of interest across the 1000 replications for available case analysis (ACA) and the 8 multiple imputation (MI) approaches under two scenarios for missing data proportions at waves 2, 4 and 6 (10, 15, 20 and 20%, 30, 40%, respectively) and four ICC combinations when data are missing completely at random (MCAR) and missing at random (MAR-inflated) respectively. **Fig. S3 and S4.** shows the empirical standard errors (filled circles with error bars showing ±1.96× Monte Carlo standard errors) and average model-based standard errors (hollow circles) from 1000 replications, for available case analysis (ACA) and the 8 multiple imputation (MI) approaches under two scenarios for missing data proportions at waves 2,4 and 6 (10, 15, 20 and 20%, 30, 40%, respectively) and four ICC combinations when data are missing completely at random (MCAR) and missing at random (MAR-inflated) respectively. **Fig. S5 and S6.** shows the estimated bias in the variance components at level 1, 2 and 3 across the 1000 replications available case analysis (ACA) and the 8 multiple imputation (MI) approaches under two scenarios for missing data proportions at waves 2, 4 and 6 (10, 15, 20 and 20%, 30, 40%, respectively) and four ICC combinations when data are missing completely at random (MCAR) and missing at random (MAR-inflated) respectively.

**Additional file 2.** R syntax for the CATS data illustration

## Data Availability

The Childhood to Adolescence Transition Study (CATS) data analysed during the current study is not publicly available due to ethics requirements but are available from the corresponding author on reasonable request. Software code written for the simulation studies is available from the corresponding author on reasonable request.

## Abbreviations

ACA: Available case analysis
CATS: Childhood to Adolescence Transition Study
DI: Dummy indicators
FCS: Fully conditional specification
GLMM: Generalized linear mixed model
IRSAD: Index of Relative Socio-Economic Advantage and Disadvantage
JM: Joint modelling
LMM: Linear mixed model
MAR: Missing at random
MCAR: Missing completely at random
MI: Multiple imputation
MLMM: Multivariate linear mixed model
MNAR: Missing not at random
NAPLAN: National Assessment Program - Literacy and Numeracy
RE: Random effects
SDQ: Strengths and Difficulties Questionnaire
SE: Standard error
SEIFA: Socioeconomic Index for Areas
SES: Socio-Economic Status
SMFQ: Short Mood and Feelings Questionnaire
VCAA: Victorian Curriculum and Assessment Authority
