# Supplementary material for: Evaluation of approaches for multiple imputation of three-level data
Source: BMC Med Res Methodol. 2020 Aug 12;20:207. doi: 10.1186/s12874-020-01079-8 (PMC7422505; doi:10.1186/s12874-020-01079-8)
Supplement: Supplementary file 1 — Additional file 1: Table S1, S2 and S3. contains the parameter values used in the data generating and missing data generation models of the simulation study. Table S4, S5 and S6 contains the values of performance measures, for available case analysis (ACA) and 8 multiple imputation (MI) approaches for estimating the regression coefficient of depressive symptom scores at the previous wave, under the three missing data mechanisms missing completely at random (MCAR), missing at random similar to CATS (MAR-CATS) and inflated missing at random (MAR-inflated) respectively. Table S7 and S8. contains the values of performance measures, for ACA and 8 multiple imputation (MI) approaches in estimating the variance components at level 3, 2 and 1, when data are missing completely at random (MCAR) with low (10, 15 and 20% across waves 2, 4 and 6 respectively) and high (20, 30 and 40% across waves 2, 4 and 6 respectively missing data percentages across waves respectively. Table S9 and S10. contains the values of performance measures, for ACA and 8 multiple imputation (MI) approaches in estimating the variance components at level 3, 2 and 1, when data missing at random (MAR-CATS) with low (10, 15 and 20% across waves 2, 4 and 6 respectively) and high (20, 30 and 40% across waves 2, 4 and 6 respectively missing data percentages across waves respectively. Table S11 and S12. contains the values of performance measures, for ACA and 8 multiple imputation (MI) approaches in estimating the variance components at level 3, 2 and 1, when data are missing at random (MAR-inflated) with low (10, 15 and 20% across waves 2, 4 and 6 respectively) and high (20, 30 and 40% across waves 2, 4 and 6 respectively missing data percentages respectively. Table S13. contains the estimated regression coefficients (and standard errors) for the adjusting covariates, from available case analysis (ACA) and 8 MI approaches applied to the CATS data analysis. Fig. S1 and S2. shows the distribution of the bias in th [file 12874_2020_1079_MOESM1_ESM.docx]

Table S1: Parameter values used in the simulation study

| **Variable generated** | **Parameter** | | **Value** |
| --- | --- | --- | --- |
| Child’s age at wave 1 | Lower limit | $a$ | 7 |
|  | Upper limit | $b$ | 10 |
| Child's sex | Proportion of females | $\lambda\%$ | 0.5 |
| Child's SES quintile | SES quintile 1- cumulative proportion | $\theta_{0}$ | 0.1 |
|  | SES quintile 2-cumulative proportion | $\theta_{1}$ | 0.2 |
|  | SES quintile 3-cumulative proportion | $\theta_{2}$ | 0.4 |
|  | SES quintile 4-cumulative proportion | $\theta_{3}$ | 0.7 |
| Standardized NAPLAN numeracy scores at wave 1 | Constant | $\eta_{0}$ | -1.24 |
|  | Sex | $\eta_{1}$ | 0.22 |
|  | Age | $\eta_{2}$ | 0.09 |
|  | SES quintile 1 | $\eta_{3,0}$ | (reference) |
|  | SES quintile 2 | $\eta_{3,1}$ | -0.06 |
|  | SES quintile 3 | $\eta_{3,2}$ | 0.36 |
|  | SES quintile 4 | $\eta_{3,3}$ | 0.32 |
|  | SES quintile 5 | $\eta_{3,4}$ | 0.63 |
|  | Error term | Standard deviation $\sigma_{\psi}$ | 1 |
|  |  |  |  |
| Child's depressive symptom scores at waves 2-7 | Constant | $\delta_{0}$ | 0.7 |
|  | Age | $\delta_{1}$ | 0.1 |
|  | Sex | $\delta_{2}$ | -0.5 |
|  | NAPLAN numeracy scores at wave 1 | $\delta_{3}$ | -0.1 |
|  | SES quintile 1 | $\delta_{4,0}$ | (reference) |
|  | SES quintile 2 | $\delta_{4,1}$ | -0.04 |
|  | SES quintile 3 | $\delta_{4,2}$ | -0.3 |
|  | SES quintile 4 | $\delta_{4,3}$ | -0.47 |
|  | SES quintile 5 | $\delta_{4,4}$ | -0.47 |
|  | Wave | $\delta_{5}$ | -0.58 |
|  | Random effects- Level 3 | Standard deviation$\sigma_{u_{3}}$ | 0.1 |
|  | Level 2 | Standard deviation $\sigma_{u_{2}}$ | 0.9 |
|  | Error term | Standard deviation $\sigma_{\varphi}$ | 1.5 |
|  |  |  |  |
| Child's standardized NAPLAN numeracy scores at waves 3,5 and 7 | Constant | $\beta_{0}$ | 1.8 |
|  | Depressive symptom score at previous wave | $\beta_{1}$ | -0.025 |
|  | Wave | $\beta_{2}$ | -0.02 |
|  | Age | $\beta_{3}$ | -0.2 |
|  | Sex | $\beta_{4}$ | 0.14 |
|  | NAPLAN numeracy scores at wave 1 | $\beta_{5}$ | 0.71 |
|  | SES quintile 1 | $\beta_{6,0}$ | (reference) |
|  | SES quintile 2 | $\beta_{6,1}$ | -0.04 |
|  | SES quintile 3 | $\beta_{6,2}$ | -0.07 |
|  | SES quintile 4 | $\beta_{6,3}$ | 0.03 |
|  | SES quintile 5 | $\beta_{6,4}$ | -0.01 |
|  | Random effects-Level 3 | Standard deviation $\sigma_{3}$ | see table S2 |
|  | Level 2 | Standard deviation $\sigma_{2}$ | see table S2 |
|  | Error term | Standard deviation $\sigma_{1}$ | see table S2 |
|  |  |  |  |
| Overall child behaviour reported by SDQ at waves 2,4 and 6 | Constant | $\gamma_{0}$ | 16.2 |
|  | Depressive symptom score at concurrent wave | $\gamma_{1}$ | 0.5 |
|  | Wave | $\gamma_{2}$ | -0.1 |
|  | Random effects-Level 3 | Standard deviation $\sigma_{v_{3}}$ | 4.1 |
|  | Level 2 | Standard deviation$\sigma_{v_{2}}$ | 0.6 |
|  | Error term | Standard deviation $\sigma_{\epsilon}$ | 2.8 |
|  |  |  |  |

SES: Socio-Economic Status, NAPLAN: National Assessment Program - Literacy and Numeracy, SDQ: Strengths and Difficulties Questionnaire.

Table S2: Intra-cluster correlation (ICC) value combinations and the respective variance components used in the simulation study

|  | **Scenario** | **ICC at level 2** | **ICC at level 3** | **Level 3 variance component**  $\boldsymbol{\sigma}_{\boldsymbol{b}\mathbf{0}\boldsymbol{i}}^{\mathbf{2}}$  **(SD)** | **Level 2 variance component**  $\boldsymbol{\sigma}_{\boldsymbol{b}\mathbf{0}\boldsymbol{ij}}^{\mathbf{2}}$  **(SD)** | **Level 1 variance component**  $\boldsymbol{\sigma}_{\boldsymbol{\varepsilon}}^{\mathbf{2}}$  **(SD)** |
| --- | --- | --- | --- | --- | --- | --- |
| 1 | High-high | 0.15 | 0.5 | 0.15(0.4) | 0.35(0.6) | 0.5(0.7) |
| 2 | High-low | 0.15 | 0.2 | 0.15(0.4) | 0.05(0.2) | 0.8(0.9) |
| 3 | Low-high | 0.05 | 0.5 | 0.05(0.2) | 0.45(0.7) | 0.5(0.7) |
| 4 | Low-low | 0.05 | 0.2 | 0.05(0.2) | 0.15(0.4) | 0.8(0.9) |

SD: Standard deviation component

Table S3: Parameter values used in logistic regression to generate missing data under missing at random (MAR) scenarios

|  | **MAR -CATS** | **MAR–inflated** |
| --- | --- | --- |
|  | **Odds ratio**  **exp(**$\boldsymbol{\zeta}_{\boldsymbol{i}}\boldsymbol{)}$ | **Odds ratio**  **exp(**$\boldsymbol{\zeta}_{\boldsymbol{i}}\boldsymbol{)}$ |
| Standardised NAPLAN numeracy scores $({NAPLAN\_z}_{ij(k+1)})$ | 1.5 | 2.0 |
| SDQ measure (${SDQ}_{ijk})$ | 3.0 | 4.0 |

NAPLAN: National Assessment Program - Literacy and Numeracy, SDQ: Strengths and Difficulties Questionnaire

Table S4: Performance of the available case analysis (ACA) and the 8 multiple imputation (MI) methods for estimating the regression coefficient of depressive symptom scores at the previous wave $\left( \beta_{1}= -0.025 \right)$ when missing data are missing completely at random (MCAR)

| **Missing data proportion at wave 2 (10%), 4(15%) and 6(20%)** | | | | | | **Missing data proportion at wave 2 (20%), 4(30%) and 6(40%)** | | | | | |
| --- | --- | --- | --- | --- | --- | --- | --- | --- | --- | --- | --- |
| **ICC combination**  **(ICC at level 3- ICC at level 2)** | **Method** | **Previous wave depression** | | | | **ICC combination**  **(ICC at level 3- ICC at level 2)** | **Method** | **Previous wave depression** | | | |
|  |  | **Bias ^a^** | **Emp SE ^b^** | **Model SE ^c^** | **Coverage** |  |  | **Bias ^a^** | **Emp SE ^b^** | **Model SE ^c^** | **Coverage** |
| High-high | ACA | 0.000 | 0.009 | 0.009 | 95.10% | High-high | ACA | 0.000 | 0.010 | 0.010 | 94.40% |
|  | JM-1L-DI-wide | 0.000 | 0.009 | 0.009 | 95.20% |  | JM-1L-DI-wide | 0.001 | 0.010 | 0.010 | 95.40% |
|  | FCS-1L-DI-wide | 0.000 | 0.009 | 0.009 | 95.60% |  | FCS-1L-DI-wide | 0.001 | 0.010 | 0.010 | 95.80% |
|  | JM-2L-wide | 0.000 | 0.009 | 0.009 | 95.40% |  | JM-2L-wide | 0.001 | 0.010 | 0.010 | 94.70% |
|  | FCS-2L-wide | 0.000 | 0.009 | 0.009 | 95.40% |  | FCS-2L-wide | 0.000 | 0.010 | 0.010 | 94.60% |
|  | JM-2L-DI | 0.000 | 0.009 | 0.009 | 94.80% |  | JM-2L-DI | 0.001 | 0.010 | 0.010 | 94.50% |
|  | FCS-2L-DI | 0.000 | 0.009 | 0.009 | 95.00% |  | FCS-2L-DI | 0.000 | 0.010 | 0.010 | 94.50% |
|  | FCS-3L-ml.lmer | 0.001 | 0.008 | 0.009 | 95.60% |  | FCS-3L-ml.lmer | 0.002 | 0.010 | 0.010 | 95.40% |
|  | FCS-3L-Blimp | 0.000 | 0.009 | 0.009 | 94.80% |  | FCS-3L-Blimp | 0.001 | 0.010 | 0.010 | 95.10% |
| High-low | ACA | 0.000 | 0.010 | 0.010 | 95.10% | High-low | ACA | 0.000 | 0.011 | 0.011 | 94.30% |
|  | JM-1L-DI-wide | 0.000 | 0.009 | 0.009 | 95.20% |  | JM-1L-DI-wide | 0.001 | 0.010 | 0.011 | 94.70% |
|  | FCS-1L-DI-wide | 0.000 | 0.009 | 0.009 | 95.00% |  | FCS-1L-DI-wide | 0.001 | 0.010 | 0.011 | 94.70% |
|  | JM-2L-wide | 0.000 | 0.009 | 0.009 | 95.40% |  | JM-2L-wide | 0.001 | 0.010 | 0.011 | 94.80% |
|  | FCS-2L-wide | 0.000 | 0.009 | 0.009 | 95.00% |  | FCS-2L-wide | 0.001 | 0.011 | 0.011 | 94.30% |
|  | JM-2L-DI | 0.000 | 0.009 | 0.009 | 95.10% |  | JM-2L-DI | 0.000 | 0.011 | 0.011 | 93.60% |
|  | FCS-2L-DI | 0.000 | 0.009 | 0.009 | 95.20% |  | FCS-2L-DI | 0.000 | 0.011 | 0.011 | 93.10% |
|  | FCS-3L-ml.lmer | 0.000 | 0.009 | 0.009 | 95.20% |  | FCS-3L-ml.lmer | 0.000 | 0.011 | 0.011 | 94.20% |
|  | FCS-3L-Blimp | 0.000 | 0.009 | 0.009 | 94.90% |  | FCS-3L-Blimp | 0.000 | 0.011 | 0.011 | 92.90% |
| Low-high | ACA | 0.000 | 0.009 | 0.009 | 95.40% | Low-high | ACA | 0.000 | 0.010 | 0.010 | 94.10% |
|  | JM-1L-DI-wide | 0.000 | 0.009 | 0.009 | 95.10% |  | JM-1L-DI-wide | 0.001 | 0.010 | 0.010 | 95.40% |
|  | FCS-1L-DI-wide | 0.000 | 0.009 | 0.009 | 95.80% |  | FCS-1L-DI-wide | 0.001 | 0.010 | 0.010 | 95.40% |
|  | JM-2L-wide | 0.000 | 0.009 | 0.009 | 95.30% |  | JM-2L-wide | 0.000 | 0.010 | 0.010 | 95.00% |
|  | FCS-2L-wide | 0.000 | 0.009 | 0.009 | 95.40% |  | FCS-2L-wide | 0.000 | 0.010 | 0.010 | 94.50% |
|  | JM-2L-DI | 0.000 | 0.009 | 0.009 | 95.20% |  | JM-2L-DI | 0.001 | 0.010 | 0.010 | 94.80% |
|  | FCS-2L-DI | 0.000 | 0.009 | 0.009 | 95.30% |  | FCS-2L-DI | 0.001 | 0.010 | 0.010 | 95.40% |
|  | FCS-3L-ml.lmer | 0.001 | 0.009 | 0.009 | 95.90% |  | FCS-3L-ml.lmer | 0.002 | 0.010 | 0.010 | 95.40% |
|  | FCS-3L-Blimp | 0.000 | 0.009 | 0.009 | 95.00% |  | FCS-3L-Blimp | 0.001 | 0.010 | 0.010 | 95.00% |
| Low-low | ACA | 0.000 | 0.010 | 0.010 | 95.10% | Low-low | ACA | 0.000 | 0.011 | 0.011 | 94.00% |
|  | JM-1L-DI-wide | 0.000 | 0.010 | 0.010 | 95.50% |  | JM-1L-DI-wide | 0.001 | 0.011 | 0.011 | 95.00% |
|  | FCS-1L-DI-wide | 0.000 | 0.010 | 0.010 | 95.40% |  | FCS-1L-DI-wide | 0.001 | 0.011 | 0.011 | 95.20% |
|  | JM-2L-wide | 0.000 | 0.010 | 0.010 | 95.30% |  | JM-2L-wide | 0.001 | 0.011 | 0.011 | 94.40% |
|  | FCS-2L-wide | 0.000 | 0.010 | 0.010 | 95.30% |  | FCS-2L-wide | 0.000 | 0.011 | 0.011 | 94.50% |
|  | JM-2L-DI | 0.000 | 0.010 | 0.010 | 95.10% |  | JM-2L-DI | 0.000 | 0.011 | 0.011 | 93.30% |
|  | FCS-2L-DI | 0.000 | 0.010 | 0.010 | 95.10% |  | FCS-2L-DI | 0.000 | 0.011 | 0.011 | 94.50% |
|  | FCS-3L-ml.lmer | 0.001 | 0.010 | 0.010 | 95.00% |  | FCS-3L-ml.lmer | 0.001 | 0.011 | 0.011 | 94.50% |
|  | FCS-3L-Blimp | 0.000 | 0.010 | 0.010 | 95.20% |  | FCS-3L-Blimp | 0.000 | 0.011 | 0.011 | 93.90% |

^a^ Estimated bias (The average of the difference between the estimate and true value across the 1000 replications) ^b^ Empirical standard error from the 1000 estimated regression coefficients ^c^Average of the model based standard error across the 1000 replications)

The following abbreviations are used to denote different MI methods, e.g., DI: dummy indicators, FCS: fully conditional specification, wide: repeated measures imputed in wide format, JM: joint modelling.

Table S5: Performance of the available case analysis (ACA) and the 8 multiple imputation methods (MI) for estimating the regression coefficient of depressive symptom scores at the previous wave $\left( \beta_{1}= -0.025 \right)$ when missing data are missing at random (MAR-CATS)

| **Missing data proportion at wave 2 (10%), 4(15%) and 6(20%)** | | | | | | **Missing data proportion at wave 2 (20%), 4(30%) and 6(40%)** | | | | | |
| --- | --- | --- | --- | --- | --- | --- | --- | --- | --- | --- | --- |
| **ICC combination**  **(ICC at level 3- ICC at level 2)** | **Method** | **Previous wave depression** | | | | **ICC combination**  **(ICC at level 3- ICC at level 2)** | **Method** | **Previous wave depression** | | | |
|  |  | **Bias ^a^** | **Emp SE ^b^** | **Model SE ^c^** | **Coverage** |  |  | **Bias ^a^** | **Emp SE ^b^** | **Model SE ^c^** | **Coverage** |
| High-high | ACA | -0.007 | 0.010 | 0.010 | 88.10% | High-high | ACA | -0.011 | 0.011 | 0.011 | 82.50% |
|  | JM-1L-DI-wide | 0.000 | 0.009 | 0.009 | 95.00% |  | JM-1L-DI-wide | 0.000 | 0.009 | 0.009 | 95.10% |
|  | FCS-1L-DI-wide | 0.000 | 0.009 | 0.009 | 94.80% |  | FCS-1L-DI-wide | 0.001 | 0.010 | 0.010 | 94.90% |
|  | JM-2L-wide | 0.000 | 0.009 | 0.009 | 94.60% |  | JM-2L-wide | 0.000 | 0.010 | 0.010 | 94.90% |
|  | FCS-2L-wide | 0.000 | 0.009 | 0.009 | 94.90% |  | FCS-2L-wide | 0.000 | 0.010 | 0.010 | 94.70% |
|  | JM-2L-DI | 0.000 | 0.009 | 0.009 | 94.90% |  | JM-2L-DI | 0.000 | 0.010 | 0.010 | 94.60% |
|  | FCS-2L-DI | 0.000 | 0.009 | 0.009 | 95.20% |  | FCS-2L-DI | 0.000 | 0.010 | 0.010 | 95.10% |
|  | FCS-3L-ml.lmer | 0.001 | 0.009 | 0.009 | 95.00% |  | FCS-3L-ml.lmer | 0.001 | 0.009 | 0.010 | 95.30% |
|  | FCS-3L-Blimp | 0.000 | 0.009 | 0.009 | 95.10% |  | FCS-3L-Blimp | 0.000 | 0.010 | 0.010 | 95.10% |
| High-low | ACA | -0.009 | 0.010 | 0.010 | 86.80% | High-low | ACA | -0.016 | 0.013 | 0.012 | 74.50% |
|  | JM-1L-DI-wide | 0.000 | 0.010 | 0.009 | 94.80% |  | JM-1L-DI-wide | 0.000 | 0.010 | 0.009 | 94.40% |
|  | FCS-1L-DI-wide | 0.000 | 0.010 | 0.009 | 94.70% |  | FCS-1L-DI-wide | 0.000 | 0.010 | 0.009 | 94.70% |
|  | JM-2L-wide | 0.000 | 0.010 | 0.009 | 94.80% |  | JM-2L-wide | 0.000 | 0.010 | 0.009 | 94.20% |
|  | FCS-2L-wide | 0.000 | 0.010 | 0.009 | 94.90% |  | FCS-2L-wide | 0.000 | 0.010 | 0.009 | 95.00% |
|  | JM-2L-DI | 0.001 | 0.009 | 0.009 | 94.80% |  | JM-2L-DI | 0.001 | 0.009 | 0.009 | 93.80% |
|  | FCS-2L-DI | 0.001 | 0.009 | 0.009 | 94.50% |  | FCS-2L-DI | 0.001 | 0.009 | 0.009 | 93.80% |
|  | FCS-3L-ml.lmer | 0.000 | 0.010 | 0.009 | 94.40% |  | FCS-3L-ml.lmer | 0.000 | 0.010 | 0.009 | 93.40% |
|  | FCS-3L-Blimp | 0.001 | 0.010 | 0.009 | 94.70% |  | FCS-3L-Blimp | 0.001 | 0.009 | 0.009 | 94.50% |
| Low-high | ACA | -0.008 | 0.010 | 0.010 | 88.00% | Low-high | ACA | -0.011 | 0.011 | 0.011 | 82.90% |
|  | JM-1L-DI-wide | 0.000 | 0.009 | 0.009 | 95.60% |  | JM-1L-DI-wide | 0.001 | 0.010 | 0.010 | 94.80% |
|  | FCS-1L-DI-wide | 0.000 | 0.009 | 0.009 | 94.90% |  | FCS-1L-DI-wide | 0.001 | 0.010 | 0.010 | 94.70% |
|  | JM-2L-wide | 0.000 | 0.009 | 0.009 | 95.00% |  | JM-2L-wide | 0.000 | 0.010 | 0.010 | 94.50% |
|  | FCS-2L-wide | 0.000 | 0.009 | 0.009 | 94.70% |  | FCS-2L-wide | 0.000 | 0.010 | 0.010 | 95.00% |
|  | JM-2L-DI | 0.000 | 0.009 | 0.009 | 95.00% |  | JM-2L-DI | 0.001 | 0.010 | 0.010 | 95.10% |
|  | FCS-2L-DI | 0.000 | 0.009 | 0.009 | 95.00% |  | FCS-2L-DI | 0.000 | 0.010 | 0.010 | 95.30% |
|  | FCS-3L-ml.lmer | 0.001 | 0.009 | 0.009 | 94.90% |  | FCS-3L-ml.lmer | 0.001 | 0.009 | 0.010 | 95.20% |
|  | FCS-3L-Blimp | 0.000 | 0.009 | 0.009 | 94.70% |  | FCS-3L-Blimp | 0.000 | 0.010 | 0.010 | 94.60% |
| Low-low | ACA | -0.010 | 0.011 | 0.011 | 85.50% | Low-low | ACA | -0.014 | 0.012 | 0.012 | 80.10% |
|  | JM-1L-DI-wide | 0.000 | 0.010 | 0.010 | 94.60% |  | JM-1L-DI-wide | 0.001 | 0.011 | 0.011 | 94.70% |
|  | FCS-1L-DI-wide | 0.000 | 0.010 | 0.010 | 94.80% |  | FCS-1L-DI-wide | 0.001 | 0.011 | 0.011 | 95.50% |
|  | JM-2L-wide | 0.000 | 0.010 | 0.010 | 94.60% |  | JM-2L-wide | 0.000 | 0.011 | 0.011 | 95.10% |
|  | FCS-2L-wide | 0.000 | 0.010 | 0.010 | 94.30% |  | FCS-2L-wide | 0.000 | 0.011 | 0.011 | 94.90% |
|  | JM-2L-DI | 0.001 | 0.010 | 0.010 | 94.40% |  | JM-2L-DI | 0.001 | 0.011 | 0.011 | 95.00% |
|  | FCS-2L-DI | 0.001 | 0.010 | 0.010 | 93.90% |  | FCS-2L-DI | 0.001 | 0.011 | 0.011 | 94.80% |
|  | FCS-3L-ml.lmer | 0.000 | 0.010 | 0.010 | 94.90% |  | FCS-3L-ml.lmer | 0.001 | 0.011 | 0.011 | 95.50% |
|  | FCS-3L-Blimp | 0.001 | 0.010 | 0.010 | 94.10% |  | FCS-3L-Blimp | 0.001 | 0.011 | 0.011 | 95.00% |

^a^ Estimated bias (The average of the difference between the estimate and true value across the 1000 replications) ^b^ Empirical standard error from the 1000 estimated regression coefficients ^c^Average of the model based standard error across the 1000 replications)

The following abbreviations are used to denote different MI methods, e.g., DI: dummy indicators, FCS: fully conditional specification, wide: repeated measures imputed in wide format, JM: joint modelling.

Table S6: Performance of the available case analysis (ACA) and the 8 multiple imputation (MI) methods for estimating the regression coefficient of depressive symptom scores at the previous wave $\left( \beta_{1}= -0.025 \right)$ when missing data are missing at random (MAR-inflated)

| **Missing data proportion at wave 2 (10%), 4(15%) and 6(20%)** | | | | | | **Missing data proportion at wave 2 (20%), 4(30%) and 6(40%)** | | | | | |
| --- | --- | --- | --- | --- | --- | --- | --- | --- | --- | --- | --- |
| **ICC combination**  **(ICC at level 3- ICC at level 2)** | **Method** | **Previous wave depression** | | | | **ICC combination**  **(ICC at level 3- ICC at level 2)** | **Method** | **Previous wave depression** | | | |
|  |  | **Bias ^a^** | **Emp SE ^b^** | **Model SE ^c^** | **Coverage** |  |  | **Bias ^a^** | **Emp SE ^b^** | **Model SE ^c^** | **Coverage** |
| High-high | ACA | -0.012 | 0.010 | 0.010 | 76.40% | High-high | ACA | -0.017 | 0.011 | 0.011 | 63.30% |
|  | JM-1L-DI-wide | 0.000 | 0.009 | 0.009 | 94.80% |  | JM-1L-DI-wide | 0.000 | 0.010 | 0.009 | 94.30% |
|  | FCS-1L-DI-wide | 0.000 | 0.009 | 0.009 | 94.50% |  | FCS-1L-DI-wide | 0.001 | 0.010 | 0.009 | 94.30% |
|  | JM-2L-wide | 0.000 | 0.009 | 0.009 | 94.70% |  | JM-2L-wide | 0.000 | 0.010 | 0.010 | 94.50% |
|  | FCS-2L-wide | 0.000 | 0.009 | 0.009 | 94.60% |  | FCS-2L-wide | 0.000 | 0.010 | 0.010 | 94.60% |
|  | JM-2L-DI | 0.000 | 0.009 | 0.009 | 94.60% |  | JM-2L-DI | 0.001 | 0.010 | 0.010 | 95.10% |
|  | FCS-2L-DI | 0.000 | 0.009 | 0.009 | 94.10% |  | FCS-2L-DI | 0.001 | 0.010 | 0.010 | 95.10% |
|  | FCS-3L-ml.lmer | 0.001 | 0.009 | 0.009 | 94.50% |  | FCS-3L-ml.lmer | 0.001 | 0.009 | 0.010 | 95.60% |
|  | FCS-3L-Blimp | 0.001 | 0.009 | 0.009 | 94.10% |  | FCS-3L-Blimp | 0.001 | 0.010 | 0.010 | 94.60% |
| High-low | ACA | -0.013 | 0.011 | 0.010 | 74.50% | High-low | ACA | -0.019 | 0.012 | 0.011 | 61.70% |
|  | JM-1L-DI-wide | 0.000 | 0.010 | 0.010 | 94.70% |  | JM-1L-DI-wide | 0.001 | 0.010 | 0.010 | 94.50% |
|  | FCS-1L-DI-wide | 0.000 | 0.010 | 0.010 | 94.40% |  | FCS-1L-DI-wide | 0.001 | 0.010 | 0.010 | 94.80% |
|  | JM-2L-wide | 0.000 | 0.010 | 0.010 | 94.50% |  | JM-2L-wide | 0.001 | 0.010 | 0.010 | 94.90% |
|  | FCS-2L-wide | 0.000 | 0.010 | 0.010 | 94.20% |  | FCS-2L-wide | 0.000 | 0.010 | 0.010 | 94.70% |
|  | JM-2L-DI | 0.001 | 0.010 | 0.009 | 94.80% |  | JM-2L-DI | 0.002 | 0.010 | 0.010 | 94.10% |
|  | FCS-2L-DI | 0.001 | 0.010 | 0.009 | 94.00% |  | FCS-2L-DI | 0.002 | 0.010 | 0.010 | 94.30% |
|  | FCS-3L-ml.lmer | 0.000 | 0.010 | 0.010 | 94.10% |  | FCS-3L-ml.lmer | 0.000 | 0.010 | 0.010 | 93.60% |
|  | FCS-3L-Blimp | 0.001 | 0.010 | 0.009 | 94.50% |  | FCS-3L-Blimp | 0.002 | 0.010 | 0.010 | 94.50% |
| Low-high | ACA | -0.012 | 0.010 | 0.010 | 75.50% | Low-high | ACA | -0.018 | 0.011 | 0.011 | 63.00% |
|  | JM-1L-DI-wide | 0.000 | 0.009 | 0.009 | 95.10% |  | JM-1L-DI-wide | 0.001 | 0.010 | 0.010 | 94.50% |
|  | FCS-1L-DI-wide | 0.000 | 0.009 | 0.009 | 95.00% |  | FCS-1L-DI-wide | 0.001 | 0.010 | 0.010 | 94.10% |
|  | JM-2L-wide | 0.000 | 0.009 | 0.009 | 95.00% |  | JM-2L-wide | 0.000 | 0.010 | 0.010 | 94.60% |
|  | FCS-2L-wide | 0.000 | 0.009 | 0.009 | 94.70% |  | FCS-2L-wide | 0.000 | 0.010 | 0.010 | 94.40% |
|  | JM-2L-DI | 0.001 | 0.009 | 0.009 | 94.30% |  | JM-2L-DI | 0.001 | 0.010 | 0.010 | 94.90% |
|  | FCS-2L-DI | 0.001 | 0.009 | 0.009 | 94.80% |  | FCS-2L-DI | 0.001 | 0.010 | 0.010 | 94.40% |
|  | FCS-3L-ml.lmer | 0.001 | 0.009 | 0.009 | 95.10% |  | FCS-3L-ml.lmer | 0.001 | 0.009 | 0.010 | 95.40% |
|  | FCS-3L-Blimp | 0.001 | 0.009 | 0.009 | 94.70% |  | FCS-3L-Blimp | 0.001 | 0.010 | 0.010 | 94.60% |
| Low-low | ACA | -0.015 | 0.011 | 0.011 | 72.00% | Low-low | ACA | -0.021 | 0.012 | 0.012 | 57.60% |
|  | JM-1L-DI-wide | 0.000 | 0.010 | 0.010 | 94.80% |  | JM-1L-DI-wide | 0.001 | 0.011 | 0.011 | 94.30% |
|  | FCS-1L-DI-wide | 0.000 | 0.010 | 0.010 | 94.80% |  | FCS-1L-DI-wide | 0.001 | 0.011 | 0.011 | 94.60% |
|  | JM-2L-wide | 0.000 | 0.010 | 0.010 | 95.10% |  | JM-2L-wide | 0.000 | 0.011 | 0.011 | 93.60% |
|  | FCS-2L-wide | 0.000 | 0.010 | 0.010 | 95.10% |  | FCS-2L-wide | 0.000 | 0.011 | 0.011 | 94.70% |
|  | JM-2L-DI | 0.001 | 0.010 | 0.010 | 94.50% |  | JM-2L-DI | 0.002 | 0.011 | 0.011 | 93.90% |
|  | FCS-2L-DI | 0.001 | 0.010 | 0.010 | 94.40% |  | FCS-2L-DI | 0.002 | 0.011 | 0.011 | 94.20% |
|  | FCS-3L-ml.lmer | 0.000 | 0.010 | 0.010 | 94.40% |  | FCS-3L-ml.lmer | 0.001 | 0.011 | 0.011 | 94.60% |
|  | FCS-3L-Blimp | 0.001 | 0.010 | 0.010 | 94.40% |  | FCS-3L-Blimp | 0.002 | 0.011 | 0.011 | 94.60% |

^a^ Estimated bias (The average of the difference between the estimate and true value across the 1000 replications) ^b^ Empirical standard error from the 1000 estimated regression coefficients ^c^Average of the model based standard error across the 1000 replications)

The following abbreviations are used to denote different MI methods, e.g., DI: dummy indicators, FCS: fully conditional specification, wide: repeated measures imputed in wide format, JM: joint modelling.

Table S7 : Performance of the available case analysis (ACA) and the 8 multiple imputation (MI) methods in estimating the variance components (VC) at level 3, 2 and 1 with low missing data proportions (10%, 15%, and 20% at waves 2, 4 and 6 respectively) and missing data are missing completely at random (MCAR)

|  |  | **VC at level 3** | | | **VC at level 2** | | | **VC at level 3** | | |
| --- | --- | --- | --- | --- | --- | --- | --- | --- | --- | --- |
| **ICC combination**  **(ICC at level 3- ICC at level 2)** | **Method** | **Bias^a^** | **RB(%)^b^** | **Emp^c^**  **SE** | **Bias^a^** | **RB(%)^b^** | **Emp^c^**  **SE** | **Bias^a^** | **RB(%)^b^** | **Emp^c^**  **SE** |
| High-high | ACA | 0.000 | 0.268 | 0.039 | -0.001 | -0.367 | 0.024 | 0.000 | 0.027 | 0.017 |
|  | JM-1L-DI-wide | 0.001 | 0.432 | 0.039 | -0.001 | -0.150 | 0.022 | 0.000 | -0.027 | 0.015 |
|  | FCS-1L-DI-wide | 0.001 | 0.380 | 0.039 | -0.001 | -0.152 | 0.022 | 0.000 | -0.025 | 0.015 |
|  | JM-2L-wide | 0.001 | 0.377 | 0.039 | -0.001 | -0.144 | 0.022 | 0.000 | -0.027 | 0.015 |
|  | FCS-2L-wide | 0.001 | 0.368 | 0.039 | -0.001 | -0.146 | 0.022 | 0.000 | -0.027 | 0.015 |
|  | JM-2L-DI | 0.001 | 0.379 | 0.039 | -0.001 | -0.149 | 0.022 | 0.000 | -0.028 | 0.015 |
|  | FCS-2L-DI | 0.001 | 0.355 | 0.039 | -0.001 | -0.148 | 0.022 | 0.000 | -0.025 | 0.015 |
|  | FCS-3L-ml.lmer | 0.001 | 0.381 | 0.039 | -0.001 | -0.182 | 0.022 | 0.000 | -0.005 | 0.015 |
|  | FCS-3L-Blimp | 0.001 | 0.375 | 0.039 | -0.001 | -0.153 | 0.022 | 0.000 | -0.027 | 0.015 |
| High-low | ACA | 0.000 | 0.153 | 0.037 | -0.001 | -2.261 | 0.018 | 0.000 | 0.022 | 0.026 |
|  | JM-1L-DI-wide | 0.000 | 0.318 | 0.037 | 0.000 | -0.794 | 0.015 | 0.000 | -0.025 | 0.023 |
|  | FCS-1L-DI-wide | 0.000 | 0.317 | 0.037 | 0.000 | -0.817 | 0.015 | 0.000 | -0.023 | 0.023 |
|  | JM-2L-wide | 0.000 | 0.299 | 0.037 | 0.000 | -0.739 | 0.015 | 0.000 | -0.025 | 0.023 |
|  | FCS-2L-wide | 0.000 | 0.300 | 0.037 | 0.000 | -0.755 | 0.015 | 0.000 | -0.025 | 0.023 |
|  | JM-2L-DI | 0.000 | 0.315 | 0.037 | 0.000 | -0.567 | 0.015 | 0.000 | -0.038 | 0.023 |
|  | FCS-2L-DI | 0.000 | 0.315 | 0.037 | 0.000 | -0.546 | 0.015 | 0.000 | -0.040 | 0.023 |
|  | FCS-3L-ml.lmer | 0.000 | 0.319 | 0.037 | -0.001 | -1.057 | 0.015 | 0.000 | -0.010 | 0.023 |
|  | FCS-3L-Blimp | 0.000 | 0.305 | 0.037 | 0.000 | -0.558 | 0.015 | 0.000 | -0.038 | 0.023 |
| Low-high | ACA | 0.000 | 0.611 | 0.017 | -0.001 | -0.308 | 0.028 | 0.000 | 0.026 | 0.017 |
|  | JM-1L-DI-wide | 0.000 | 0.803 | 0.016 | -0.001 | -0.133 | 0.026 | 0.000 | -0.027 | 0.015 |
|  | FCS-1L-DI-wide | 0.000 | 0.800 | 0.016 | -0.001 | -0.135 | 0.026 | 0.000 | -0.025 | 0.015 |
|  | JM-2L-wide | 0.000 | 0.788 | 0.016 | -0.001 | -0.132 | 0.026 | 0.000 | -0.027 | 0.015 |
|  | FCS-2L-wide | 0.000 | 0.785 | 0.016 | -0.001 | -0.132 | 0.026 | 0.000 | -0.028 | 0.015 |
|  | JM-2L-DI | 0.000 | 0.800 | 0.016 | -0.001 | -0.138 | 0.026 | 0.000 | -0.023 | 0.015 |
|  | FCS-2L-DI | 0.000 | 0.802 | 0.016 | -0.001 | -0.139 | 0.026 | 0.000 | -0.025 | 0.015 |
|  | FCS-3L-ml.lmer | 0.000 | 0.802 | 0.016 | -0.001 | -0.158 | 0.026 | 0.000 | -0.006 | 0.015 |
|  | FCS-3L-Blimp | 0.000 | 0.783 | 0.016 | -0.001 | -0.138 | 0.026 | 0.000 | -0.025 | 0.015 |
| Low-low | ACA | 0.000 | 0.485 | 0.015 | -0.001 | -0.967 | 0.022 | 0.000 | 0.028 | 0.027 |
|  | JM-1L-DI-wide | 0.000 | 0.720 | 0.015 | -0.001 | -0.338 | 0.019 | 0.000 | -0.025 | 0.023 |
|  | FCS-1L-DI-wide | 0.000 | 0.719 | 0.015 | -0.001 | -0.346 | 0.019 | 0.000 | -0.024 | 0.023 |
|  | JM-2L-wide | 0.000 | 0.701 | 0.015 | 0.000 | -0.332 | 0.019 | 0.000 | -0.025 | 0.023 |
|  | FCS-2L-wide | 0.000 | 0.700 | 0.015 | -0.001 | -0.335 | 0.019 | 0.000 | -0.025 | 0.023 |
|  | JM-2L-DI | 0.000 | 0.716 | 0.015 | 0.000 | -0.279 | 0.019 | 0.000 | -0.035 | 0.023 |
|  | FCS-2L-DI | 0.000 | 0.715 | 0.015 | 0.000 | -0.275 | 0.019 | 0.000 | -0.036 | 0.023 |
|  | FCS-3L-ml.lmer | 0.000 | 0.719 | 0.015 | -0.001 | -0.423 | 0.019 | 0.000 | -0.011 | 0.023 |
|  | FCS-3L-Blimp | 0.000 | 0.700 | 0.015 | 0.000 | -0.276 | 0.019 | 0.000 | -0.037 | 0.023 |

^a^ Estimated bias (The average of the difference between the estimate and true value across the 1000 replications) ^b^ Empirical standard error from the 1000 estimated regression coefficients ^c^Average of the model based standard error across the 1000 simulated datasets

The following abbreviations are used to denote different MI methods, e.g., DI: dummy indicators, FCS: fully conditional specification, wide: repeated measures imputed in wide format,JM: joint modelling.

Table S8: Performance of the available case analysis (ACA) and the 8 multiple imputation (MI) methods in estimating the variance components (VC) at level 3, 2 and 1 with high missing data proportions (20%, 30%, and 40% at waves 2, 4 and 6 respectively) and missing data are missing completely at random (MCAR)

|  |  | **VC at level 3** | | | **VC at level 2** | | | **VC at level 3** | | |
| --- | --- | --- | --- | --- | --- | --- | --- | --- | --- | --- |
| **ICC combination**  **(ICC at level 3- ICC at level 2)** | **Method** | **Bias^a^** | **RB(%)^b^** | **Emp^c^**  **SE** | **Bias^a^** | **RB(%)^b^** | **Emp^c^**  **SE** | **Bias^a^** | **RB(%)^b^** | **Emp^c^**  **SE** |
| High-high | ACA | 0.002 | 1.318 | 0.039 | 0.000 | -0.055 | 0.027 | 0.001 | 0.261 | 0.019 |
|  | JM-1L-DI-wide | 0.002 | 1.596 | 0.039 | 0.000 | 0.022 | 0.023 | 0.000 | 0.094 | 0.014 |
|  | FCS-1L-DI-wide | 0.002 | 1.596 | 0.039 | 0.000 | 0.016 | 0.023 | 0.000 | 0.096 | 0.014 |
|  | JM-2L-wide | 0.002 | 1.540 | 0.039 | 0.000 | 0.049 | 0.023 | 0.000 | 0.087 | 0.014 |
|  | FCS-2L-wide | 0.002 | 1.552 | 0.039 | 0.000 | 0.040 | 0.023 | 0.000 | 0.087 | 0.014 |
|  | JM-2L-DI | 0.002 | 1.600 | 0.039 | 0.000 | -0.039 | 0.023 | 0.001 | 0.120 | 0.014 |
|  | FCS-2L-DI | 0.002 | 1.598 | 0.039 | 0.000 | -0.040 | 0.023 | 0.001 | 0.114 | 0.014 |
|  | FCS-3L-ml.lmer | 0.002 | 1.587 | 0.039 | 0.000 | -0.053 | 0.023 | 0.001 | 0.122 | 0.014 |
|  | FCS-3L-Blimp | 0.002 | 1.538 | 0.039 | 0.000 | -0.033 | 0.023 | 0.001 | 0.127 | 0.014 |
| High-low | ACA | 0.002 | 1.322 | 0.038 | 0.000 | -0.651 | 0.022 | 0.002 | 0.261 | 0.030 |
|  | JM-1L-DI-wide | 0.002 | 1.577 | 0.037 | 0.000 | -0.104 | 0.016 | 0.001 | 0.098 | 0.022 |
|  | FCS-1L-DI-wide | 0.002 | 1.577 | 0.037 | 0.000 | -0.142 | 0.016 | 0.001 | 0.099 | 0.022 |
|  | JM-2L-wide | 0.002 | 1.497 | 0.037 | 0.000 | 0.140 | 0.016 | 0.001 | 0.093 | 0.022 |
|  | FCS-2L-wide | 0.002 | 1.503 | 0.037 | 0.000 | 0.082 | 0.016 | 0.001 | 0.092 | 0.022 |
|  | JM-2L-DI | 0.002 | 1.577 | 0.037 | 0.000 | 0.347 | 0.016 | 0.001 | 0.064 | 0.022 |
|  | FCS-2L-DI | 0.002 | 1.578 | 0.037 | 0.000 | 0.365 | 0.016 | 0.000 | 0.061 | 0.022 |
|  | FCS-3L-ml.lmer | 0.002 | 1.574 | 0.037 | 0.000 | -0.593 | 0.016 | 0.001 | 0.118 | 0.022 |
|  | FCS-3L-Blimp | 0.002 | 1.524 | 0.037 | 0.000 | 0.339 | 0.016 | 0.001 | 0.071 | 0.022 |
| Low-high | ACA | 0.001 | 1.208 | 0.017 | 0.000 | -0.045 | 0.031 | 0.001 | 0.261 | 0.019 |
|  | JM-1L-DI-wide | 0.001 | 1.751 | 0.017 | 0.000 | 0.019 | 0.027 | 0.000 | 0.093 | 0.014 |
|  | FCS-1L-DI-wide | 0.001 | 1.754 | 0.017 | 0.000 | 0.015 | 0.027 | 0.000 | 0.095 | 0.014 |
|  | JM-2L-wide | 0.001 | 1.680 | 0.017 | 0.000 | 0.030 | 0.027 | 0.000 | 0.087 | 0.014 |
|  | FCS-2L-wide | 0.001 | 1.695 | 0.017 | 0.000 | 0.024 | 0.027 | 0.000 | 0.086 | 0.014 |
|  | JM-2L-DI | 0.001 | 1.763 | 0.017 | 0.000 | -0.049 | 0.027 | 0.001 | 0.134 | 0.014 |
|  | FCS-2L-DI | 0.001 | 1.761 | 0.017 | 0.000 | -0.051 | 0.027 | 0.001 | 0.130 | 0.014 |
|  | FCS-3L-ml.lmer | 0.001 | 1.722 | 0.017 | 0.000 | -0.042 | 0.027 | 0.001 | 0.121 | 0.014 |
|  | FCS-3L-Blimp | 0.001 | 1.691 | 0.017 | 0.000 | -0.048 | 0.027 | 0.001 | 0.136 | 0.014 |
| Low-low | ACA | 0.001 | 1.233 | 0.016 | 0.000 | -0.248 | 0.026 | 0.002 | 0.268 | 0.030 |
|  | JM-1L-DI-wide | 0.001 | 1.856 | 0.015 | 0.000 | 0.013 | 0.020 | 0.001 | 0.095 | 0.022 |
|  | FCS-1L-DI-wide | 0.001 | 1.860 | 0.015 | 0.000 | 0.000 | 0.020 | 0.001 | 0.096 | 0.022 |
|  | JM-2L-wide | 0.001 | 1.762 | 0.015 | 0.000 | 0.051 | 0.020 | 0.001 | 0.091 | 0.022 |
|  | FCS-2L-wide | 0.001 | 1.773 | 0.015 | 0.000 | 0.033 | 0.020 | 0.001 | 0.090 | 0.022 |
|  | JM-2L-DI | 0.001 | 1.857 | 0.015 | 0.000 | 0.088 | 0.020 | 0.001 | 0.075 | 0.022 |
|  | FCS-2L-DI | 0.001 | 1.861 | 0.015 | 0.000 | 0.092 | 0.020 | 0.001 | 0.073 | 0.022 |
|  | FCS-3L-ml.lmer | 0.001 | 1.839 | 0.015 | 0.000 | -0.157 | 0.020 | 0.001 | 0.114 | 0.022 |
|  | FCS-3L-Blimp | 0.001 | 1.794 | 0.015 | 0.000 | 0.088 | 0.020 | 0.001 | 0.076 | 0.022 |

^a^ Estimated bias (The average of the difference between the estimate and true value across the 1000 simulated datasets) ^b^ Empirical standard error from the 1000 estimated regression coefficients ^c^Average of the model based standard error across the 1000 replications

The following abbreviations are used to denote different MI methods, e.g., DI: dummy indicators, FCS: fully conditional specification,wide: repeated measures imputed in wide format,JM: joint modelling.

Table S9: Performance of the available case analysis (ACA) and the 8 multiple imputation (MI) methods in estimating the variance components (VC) at level 3, 2 and 1 with low missing data proportions (10%, 15%, and 20% at waves 2, 4 and 6 respectively) and missing data are missing at random (MAR)

|  |  | **VC at level 3** | | | **VC at level 2** | | | **VC at level 3** | | |
| --- | --- | --- | --- | --- | --- | --- | --- | --- | --- | --- |
| **ICC combination**  **(ICC at level 3- ICC at level 2)** | **Method** | **Bias^a^** | **RB(%)^b^** | **Emp^c^**  **SE** | **Bias^a^** | **RB(%)^b^** | **Emp^c^**  **SE** | **Bias^a^** | **RB(%)^b^** | **Emp^c^**  **SE** |
| High-high | ACA | 0.001 | 0.750 | 0.039 | -0.001 | -0.400 | 0.025 | 0.000 | -0.052 | 0.016 |
|  | JM-1L-DI-wide | 0.002 | 1.575 | 0.039 | 0.000 | 0.026 | 0.023 | 0.001 | 0.107 | 0.014 |
|  | FCS-1L-DI-wide | 0.002 | 1.577 | 0.039 | 0.000 | 0.021 | 0.023 | 0.001 | 0.106 | 0.014 |
|  | JM-2L-wide | 0.002 | 1.559 | 0.039 | 0.000 | 0.031 | 0.023 | 0.001 | 0.106 | 0.014 |
|  | FCS-2L-wide | 0.002 | 1.559 | 0.039 | 0.000 | 0.028 | 0.023 | 0.001 | 0.107 | 0.014 |
|  | JM-2L-DI | 0.002 | 1.595 | 0.039 | 0.000 | 0.055 | 0.023 | 0.001 | 0.105 | 0.014 |
|  | FCS-2L-DI | 0.002 | 1.594 | 0.039 | 0.000 | 0.055 | 0.023 | 0.001 | 0.105 | 0.014 |
|  | FCS-3L-ml.lmer | 0.002 | 1.573 | 0.039 | 0.000 | -0.008 | 0.023 | 0.001 | 0.126 | 0.014 |
|  | FCS-3L-Blimp | 0.002 | 1.580 | 0.039 | 0.000 | 0.051 | 0.023 | 0.001 | 0.108 | 0.014 |
| High-low | ACA | 0.001 | 0.745 | 0.038 | 0.000 | 0.600 | 0.018 | -0.001 | -0.161 | 0.025 |
|  | JM-1L-DI-wide | 0.002 | 1.564 | 0.037 | 0.000 | -0.090 | 0.016 | 0.001 | 0.109 | 0.022 |
|  | FCS-1L-DI-wide | 0.002 | 1.559 | 0.037 | 0.000 | -0.112 | 0.016 | 0.001 | 0.109 | 0.022 |
|  | JM-2L-wide | 0.002 | 1.532 | 0.037 | 0.000 | -0.044 | 0.016 | 0.001 | 0.108 | 0.022 |
|  | FCS-2L-wide | 0.002 | 1.537 | 0.037 | 0.000 | -0.055 | 0.016 | 0.001 | 0.109 | 0.022 |
|  | JM-2L-DI | 0.002 | 1.578 | 0.037 | 0.000 | 0.298 | 0.016 | 0.001 | 0.095 | 0.022 |
|  | FCS-2L-DI | 0.002 | 1.577 | 0.037 | 0.000 | 0.296 | 0.016 | 0.001 | 0.095 | 0.022 |
|  | FCS-3L-ml.lmer | 0.002 | 1.555 | 0.037 | 0.000 | -0.352 | 0.016 | 0.001 | 0.123 | 0.022 |
|  | FCS-3L-Blimp | 0.002 | 1.563 | 0.037 | 0.000 | 0.286 | 0.016 | 0.001 | 0.097 | 0.022 |
| Low-high | ACA | 0.000 | 0.628 | 0.017 | -0.002 | -0.440 | 0.029 | 0.000 | -0.057 | 0.016 |
|  | JM-1L-DI-wide | 0.001 | 1.708 | 0.017 | 0.000 | 0.024 | 0.027 | 0.001 | 0.105 | 0.014 |
|  | FCS-1L-DI-wide | 0.001 | 1.690 | 0.017 | 0.000 | 0.019 | 0.027 | 0.001 | 0.107 | 0.014 |
|  | JM-2L-wide | 0.001 | 1.666 | 0.017 | 0.000 | 0.023 | 0.027 | 0.001 | 0.106 | 0.014 |
|  | FCS-2L-wide | 0.001 | 1.676 | 0.017 | 0.000 | 0.022 | 0.027 | 0.001 | 0.107 | 0.014 |
|  | JM-2L-DI | 0.001 | 1.718 | 0.017 | 0.000 | 0.040 | 0.027 | 0.001 | 0.109 | 0.014 |
|  | FCS-2L-DI | 0.001 | 1.719 | 0.017 | 0.000 | 0.039 | 0.027 | 0.001 | 0.109 | 0.014 |
|  | FCS-3L-ml.lmer | 0.001 | 1.689 | 0.017 | 0.000 | -0.006 | 0.027 | 0.001 | 0.126 | 0.014 |
|  | FCS-3L-Blimp | 0.001 | 1.698 | 0.017 | 0.000 | 0.046 | 0.027 | 0.001 | 0.106 | 0.014 |
| Low-low | ACA | 0.000 | 0.648 | 0.015 | 0.000 | -0.196 | 0.022 | -0.001 | -0.168 | 0.025 |
|  | JM-1L-DI-wide | 0.001 | 1.810 | 0.015 | 0.000 | 0.025 | 0.020 | 0.001 | 0.107 | 0.022 |
|  | FCS-1L-DI-wide | 0.001 | 1.809 | 0.015 | 0.000 | 0.010 | 0.020 | 0.001 | 0.108 | 0.022 |
|  | JM-2L-wide | 0.001 | 1.774 | 0.015 | 0.000 | 0.021 | 0.020 | 0.001 | 0.108 | 0.022 |
|  | FCS-2L-wide | 0.001 | 1.781 | 0.015 | 0.000 | 0.017 | 0.020 | 0.001 | 0.108 | 0.022 |
|  | JM-2L-DI | 0.001 | 1.820 | 0.015 | 0.000 | 0.141 | 0.020 | 0.001 | 0.096 | 0.022 |
|  | FCS-2L-DI | 0.001 | 1.823 | 0.015 | 0.000 | 0.141 | 0.020 | 0.001 | 0.097 | 0.022 |
|  | FCS-3L-ml.lmer | 0.001 | 1.792 | 0.015 | 0.000 | -0.068 | 0.020 | 0.001 | 0.121 | 0.022 |
|  | FCS-3L-Blimp | 0.001 | 1.802 | 0.015 | 0.000 | 0.143 | 0.020 | 0.001 | 0.096 | 0.022 |

^a^ Estimated bias (The average of the difference between the estimate and true value across the 1000 simulated datasets) ^b^ Empirical standard error from the 1000 estimated regression coefficients ^c^Average of the model based standard error across the 1000 replications

The following abbreviations are used to denote different MI methods, e.g., DI: dummy indicators, FCS: fully conditional specification,wide: repeated measures imputed in wide format,JM: joint modelling.

.

Table S10: Performance of the available case analysis (ACA) and the 8 multiple imputation (MI) methods in estimating the variance components (VC) at level 3, 2 and 1 with high missing data proportions (20%, 30%, and 40% at waves 2, 4 and 6 respectively) and missing data are missing at random (MAR)

|  |  | **VC at level 3** | | | **VC at level 2** | | | **VC at level 3** | | |
| --- | --- | --- | --- | --- | --- | --- | --- | --- | --- | --- |
| **ICC combination**  **(ICC at level 3- ICC at level 2)** | **Method** | **Bias^a^** | **RB(%)^b^** | **Emp^c^**  **SE** | **Bias^a^** | **RB(%)^b^** | **Emp^c^**  **SE** | **Bias^a^** | **RB(%)^b^** | **Emp^c^**  **SE** |
| High-high | ACA | 0.001 | 0.526 | 0.039 | -0.002 | -0.601 | 0.027 | -0.001 | -0.151 | 0.019 |
|  | JM-1L-DI-wide | 0.002 | 1.579 | 0.039 | 0.000 | 0.021 | 0.023 | 0.000 | 0.099 | 0.014 |
|  | FCS-1L-DI-wide | 0.002 | 1.575 | 0.039 | 0.000 | 0.015 | 0.023 | 0.001 | 0.102 | 0.014 |
|  | JM-2L-wide | 0.002 | 1.534 | 0.039 | 0.000 | 0.038 | 0.023 | 0.000 | 0.095 | 0.014 |
|  | FCS-2L-wide | 0.002 | 1.542 | 0.039 | 0.000 | 0.030 | 0.023 | 0.000 | 0.095 | 0.014 |
|  | JM-2L-DI | 0.002 | 1.609 | 0.039 | 0.000 | 0.060 | 0.023 | 0.000 | 0.096 | 0.014 |
|  | FCS-2L-DI | 0.002 | 1.606 | 0.039 | 0.000 | 0.060 | 0.023 | 0.000 | 0.095 | 0.014 |
|  | FCS-3L-ml.lmer | 0.002 | 1.570 | 0.039 | 0.000 | -0.035 | 0.023 | 0.001 | 0.127 | 0.014 |
|  | FCS-3L-Blimp | 0.002 | 1.564 | 0.039 | 0.000 | 0.063 | 0.023 | 0.001 | 0.100 | 0.014 |
| High-low | ACA | 0.001 | 0.636 | 0.038 | 0.001 | 1.214 | 0.021 | -0.002 | -0.242 | 0.028 |
|  | JM-1L-DI-wide | 0.002 | 1.567 | 0.037 | 0.000 | -0.090 | 0.016 | 0.001 | 0.108 | 0.022 |
|  | FCS-1L-DI-wide | 0.002 | 1.565 | 0.037 | 0.000 | -0.099 | 0.016 | 0.001 | 0.109 | 0.022 |
|  | JM-2L-wide | 0.002 | 1.540 | 0.037 | 0.000 | -0.014 | 0.016 | 0.001 | 0.108 | 0.022 |
|  | FCS-2L-wide | 0.002 | 1.548 | 0.037 | 0.000 | -0.060 | 0.016 | 0.001 | 0.108 | 0.022 |
|  | JM-2L-DI | 0.002 | 1.586 | 0.037 | 0.000 | 0.334 | 0.016 | 0.001 | 0.096 | 0.022 |
|  | FCS-2L-DI | 0.002 | 1.586 | 0.037 | 0.000 | 0.337 | 0.016 | 0.001 | 0.095 | 0.022 |
|  | FCS-3L-ml.lmer | 0.002 | 1.556 | 0.037 | 0.000 | -0.359 | 0.016 | 0.001 | 0.122 | 0.022 |
|  | FCS-3L-Blimp | 0.002 | 1.576 | 0.037 | 0.000 | 0.331 | 0.016 | 0.001 | 0.097 | 0.022 |
| Low-high | ACA | 0.000 | 0.554 | 0.017 | -0.003 | -0.667 | 0.032 | -0.001 | -0.143 | 0.019 |
|  | JM-1L-DI-wide | 0.001 | 1.715 | 0.017 | 0.000 | 0.018 | 0.027 | 0.000 | 0.099 | 0.014 |
|  | FCS-1L-DI-wide | 0.001 | 1.710 | 0.017 | 0.000 | 0.017 | 0.027 | 0.001 | 0.101 | 0.014 |
|  | JM-2L-wide | 0.001 | 1.649 | 0.017 | 0.000 | 0.028 | 0.027 | 0.000 | 0.097 | 0.014 |
|  | FCS-2L-wide | 0.001 | 1.666 | 0.017 | 0.000 | 0.021 | 0.027 | 0.000 | 0.096 | 0.014 |
|  | JM-2L-DI | 0.001 | 1.741 | 0.017 | 0.000 | 0.041 | 0.027 | 0.001 | 0.107 | 0.014 |
|  | FCS-2L-DI | 0.001 | 1.741 | 0.017 | 0.000 | 0.039 | 0.027 | 0.001 | 0.104 | 0.014 |
|  | FCS-3L-ml.lmer | 0.001 | 1.720 | 0.017 | 0.000 | -0.028 | 0.027 | 0.001 | 0.128 | 0.014 |
|  | FCS-3L-Blimp | 0.001 | 1.690 | 0.017 | 0.000 | 0.038 | 0.027 | 0.001 | 0.105 | 0.014 |
| Low-low | ACA | 0.000 | 0.563 | 0.016 | -0.001 | -0.412 | 0.026 | -0.003 | -0.337 | 0.030 |
|  | JM-1L-DI-wide | 0.001 | 1.819 | 0.015 | 0.000 | 0.010 | 0.020 | 0.001 | 0.101 | 0.022 |
|  | FCS-1L-DI-wide | 0.001 | 1.819 | 0.015 | 0.000 | -0.001 | 0.020 | 0.001 | 0.104 | 0.022 |
|  | JM-2L-wide | 0.001 | 1.740 | 0.015 | 0.000 | 0.037 | 0.020 | 0.001 | 0.100 | 0.022 |
|  | FCS-2L-wide | 0.001 | 1.758 | 0.015 | 0.000 | 0.012 | 0.020 | 0.001 | 0.099 | 0.022 |
|  | JM-2L-DI | 0.001 | 1.834 | 0.015 | 0.000 | 0.234 | 0.020 | 0.001 | 0.079 | 0.022 |
|  | FCS-2L-DI | 0.001 | 1.832 | 0.015 | 0.000 | 0.232 | 0.020 | 0.001 | 0.078 | 0.022 |
|  | FCS-3L-ml.lmer | 0.001 | 1.799 | 0.015 | 0.000 | -0.129 | 0.020 | 0.001 | 0.121 | 0.022 |
|  | FCS-3L-Blimp | 0.001 | 1.791 | 0.015 | 0.000 | 0.231 | 0.020 | 0.001 | 0.080 | 0.022 |

^a^ Estimated bias (The average of the difference between the estimate and true value across the 1000 simulated datasets) ^b^ Empirical standard error from the 1000 estimated regression coefficients ^c^Average of the model based standard error across the 1000 replications

The following abbreviations are used to denote different MI methods, e.g., DI: dummy indicators, FCS: fully conditional specification,wide: repeated measures imputed in wide format,JM: joint modelling.

Table S11: Performance of the available case analysis (ACA) and the 8 multiple imputation (MI) methods in estimating the variance components (VC) at level 3, 2 and 1 with low missing data proportions (10%, 15% and 20% at waves 2, 4 and 6 respectively) and missing data are missing at random (MAR- inflated)

|  |  | **VC at level 3** | | | **VC at level 2** | | | **VC at level 3** | | |
| --- | --- | --- | --- | --- | --- | --- | --- | --- | --- | --- |
| **ICC combination**  **(ICC at level 3- ICC at level 2)** | **Method** | **Bias^a^** | **RB(%)^b^** | **Emp^c^**  **SE** | **Bias^a^** | **RB(%)^b^** | **Emp^c^**  **SE** | **Bias^a^** | **RB(%)^b^** | **Emp^c^**  **SE** |
| High-high | ACA | 0.000 | 0.276 | 0.039 | -0.002 | -0.714 | 0.025 | -0.002 | -0.322 | 0.016 |
|  | JM-1L-DI-wide | 0.002 | 1.575 | 0.039 | 0.000 | 0.025 | 0.023 | 0.001 | 0.106 | 0.014 |
|  | FCS-1L-DI-wide | 0.002 | 1.573 | 0.039 | 0.000 | 0.021 | 0.023 | 0.001 | 0.106 | 0.014 |
|  | JM-2L-wide | 0.002 | 1.552 | 0.039 | 0.000 | 0.030 | 0.023 | 0.001 | 0.105 | 0.014 |
|  | FCS-2L-wide | 0.002 | 1.557 | 0.039 | 0.000 | 0.029 | 0.023 | 0.001 | 0.105 | 0.014 |
|  | JM-2L-DI | 0.002 | 1.609 | 0.039 | 0.000 | 0.067 | 0.023 | 0.001 | 0.105 | 0.014 |
|  | FCS-2L-DI | 0.002 | 1.608 | 0.039 | 0.000 | 0.068 | 0.023 | 0.001 | 0.105 | 0.014 |
|  | FCS-3L-ml.lmer | 0.002 | 1.570 | 0.039 | 0.000 | -0.009 | 0.023 | 0.001 | 0.125 | 0.014 |
|  | FCS-3L-Blimp | 0.002 | 1.587 | 0.039 | 0.000 | 0.067 | 0.023 | 0.001 | 0.108 | 0.014 |
| High-low | ACA | 0.000 | 0.253 | 0.037 | 0.001 | 1.855 | 0.018 | -0.004 | -0.556 | 0.025 |
|  | JM-1L-DI-wide | 0.002 | 1.561 | 0.037 | 0.000 | -0.107 | 0.016 | 0.001 | 0.107 | 0.022 |
|  | FCS-1L-DI-wide | 0.002 | 1.561 | 0.037 | 0.000 | -0.134 | 0.016 | 0.001 | 0.107 | 0.022 |
|  | JM-2L-wide | 0.002 | 1.532 | 0.037 | 0.000 | -0.032 | 0.016 | 0.001 | 0.106 | 0.022 |
|  | FCS-2L-wide | 0.002 | 1.537 | 0.037 | 0.000 | -0.061 | 0.016 | 0.001 | 0.107 | 0.023 |
|  | JM-2L-DI | 0.002 | 1.586 | 0.037 | 0.000 | 0.356 | 0.016 | 0.001 | 0.095 | 0.022 |
|  | FCS-2L-DI | 0.002 | 1.588 | 0.037 | 0.000 | 0.344 | 0.016 | 0.001 | 0.095 | 0.022 |
|  | FCS-3L-ml.lmer | 0.002 | 1.554 | 0.037 | 0.000 | -0.369 | 0.016 | 0.001 | 0.123 | 0.022 |
|  | FCS-3L-Blimp | 0.002 | 1.572 | 0.037 | 0.000 | 0.327 | 0.016 | 0.001 | 0.096 | 0.022 |
| Low-high | ACA | 0.000 | 0.182 | 0.017 | -0.004 | -0.806 | 0.029 | -0.002 | -0.313 | 0.016 |
|  | JM-1L-DI-wide | 0.001 | 1.694 | 0.017 | 0.000 | 0.023 | 0.027 | 0.001 | 0.106 | 0.014 |
|  | FCS-1L-DI-wide | 0.001 | 1.685 | 0.017 | 0.000 | 0.018 | 0.027 | 0.001 | 0.105 | 0.014 |
|  | JM-2L-wide | 0.001 | 1.666 | 0.017 | 0.000 | 0.022 | 0.027 | 0.001 | 0.106 | 0.014 |
|  | FCS-2L-wide | 0.001 | 1.675 | 0.017 | 0.000 | 0.023 | 0.027 | 0.001 | 0.105 | 0.014 |
|  | JM-2L-DI | 0.001 | 1.725 | 0.017 | 0.000 | 0.055 | 0.027 | 0.001 | 0.110 | 0.014 |
|  | FCS-2L-DI | 0.001 | 1.726 | 0.017 | 0.000 | 0.055 | 0.027 | 0.001 | 0.110 | 0.014 |
|  | FCS-3L-ml.lmer | 0.001 | 1.681 | 0.017 | 0.000 | -0.005 | 0.027 | 0.001 | 0.127 | 0.014 |
|  | FCS-3L-Blimp | 0.001 | 1.711 | 0.017 | 0.000 | 0.053 | 0.027 | 0.001 | 0.110 | 0.014 |
| Low-low | ACA | 0.000 | 0.159 | 0.015 | 0.000 | -0.168 | 0.022 | -0.004 | -0.561 | 0.025 |
|  | JM-1L-DI-wide | 0.001 | 1.802 | 0.015 | 0.000 | 0.015 | 0.020 | 0.001 | 0.107 | 0.022 |
|  | FCS-1L-DI-wide | 0.001 | 1.800 | 0.015 | 0.000 | 0.000 | 0.020 | 0.001 | 0.106 | 0.022 |
|  | JM-2L-wide | 0.001 | 1.774 | 0.015 | 0.000 | 0.012 | 0.020 | 0.001 | 0.107 | 0.022 |
|  | FCS-2L-wide | 0.001 | 1.776 | 0.015 | 0.000 | 0.012 | 0.020 | 0.001 | 0.108 | 0.022 |
|  | JM-2L-DI | 0.001 | 1.827 | 0.015 | 0.000 | 0.159 | 0.020 | 0.001 | 0.098 | 0.022 |
|  | FCS-2L-DI | 0.001 | 1.829 | 0.015 | 0.000 | 0.163 | 0.020 | 0.001 | 0.097 | 0.022 |
|  | FCS-3L-ml.lmer | 0.001 | 1.797 | 0.015 | 0.000 | -0.080 | 0.020 | 0.001 | 0.121 | 0.022 |
|  | FCS-3L-Blimp | 0.001 | 1.809 | 0.015 | 0.000 | 0.156 | 0.020 | 0.001 | 0.097 | 0.022 |

^a^ Estimated bias (The average of the difference between the estimate and true value across the 1000 simulated datasets) ^b^ Empirical standard error from the 1000 estimated regression coefficients ^c^Average of the model based standard error across the 1000 replications

The following abbreviations are used to denote different MI methods, e.g., DI: dummy indicators, FCS: fully conditional specification,wide: repeated measures imputed in wide format,JM: joint modelling.

Table S12: Performance of the available case analysis (ACA) and the 8 multiple imputation (MI) methods in estimating the variance components (VC) at level 3, 2 and 1 with high missing data proportions (20%,30% and 40% at waves 2, 4 and 6 respectively) and missing data are missing at random (MAR- inflated)

|  |  | **VC at level 3** | | | **VC at level 2** | | | **VC at level 3** | | |
| --- | --- | --- | --- | --- | --- | --- | --- | --- | --- | --- |
| **ICC combination**  **(ICC at level 3- ICC at level 2)** | **Method** | **Bias^a^** | **RB(%)^b^** | **Emp^c^**  **SE** | **Bias^a^** | **RB(%)^b^** | **Emp^c^**  **SE** | **Bias^a^** | **RB(%)^b^** | **Emp^c^**  **SE** |
| High-high | ACA | -0.001 | -0.500 | 0.039 | -0.004 | -1.127 | 0.027 | -0.002 | -0.480 | 0.018 |
|  | JM-1L-DI-wide | 0.002 | 1.578 | 0.039 | 0.000 | 0.021 | 0.023 | 0.000 | 0.100 | 0.014 |
|  | FCS-1L-DI-wide | 0.002 | 1.579 | 0.039 | 0.000 | 0.016 | 0.023 | 0.001 | 0.101 | 0.014 |
|  | JM-2L-wide | 0.002 | 1.535 | 0.039 | 0.000 | 0.042 | 0.023 | 0.000 | 0.096 | 0.014 |
|  | FCS-2L-wide | 0.002 | 1.542 | 0.039 | 0.000 | 0.032 | 0.023 | 0.000 | 0.096 | 0.014 |
|  | JM-2L-DI | 0.002 | 1.628 | 0.039 | 0.000 | 0.090 | 0.023 | 0.001 | 0.102 | 0.014 |
|  | FCS-2L-DI | 0.002 | 1.630 | 0.039 | 0.000 | 0.090 | 0.023 | 0.000 | 0.100 | 0.014 |
|  | FCS-3L-ml.lmer | 0.002 | 1.580 | 0.039 | 0.000 | -0.031 | 0.023 | 0.001 | 0.125 | 0.014 |
|  | FCS-3L-Blimp | 0.002 | 1.586 | 0.039 | 0.000 | 0.084 | 0.023 | 0.001 | 0.105 | 0.014 |
| High-low | ACA | -0.001 | -0.420 | 0.037 | 0.001 | 2.688 | 0.021 | -0.007 | -0.881 | 0.029 |
|  | JM-1L-DI-wide | 0.002 | 1.562 | 0.037 | 0.000 | -0.122 | 0.016 | 0.001 | 0.104 | 0.022 |
|  | FCS-1L-DI-wide | 0.002 | 1.557 | 0.037 | 0.000 | -0.108 | 0.016 | 0.001 | 0.103 | 0.022 |
|  | JM-2L-wide | 0.002 | 1.496 | 0.037 | 0.000 | 0.055 | 0.016 | 0.001 | 0.101 | 0.022 |
|  | FCS-2L-wide | 0.002 | 1.502 | 0.037 | 0.000 | 0.030 | 0.016 | 0.001 | 0.099 | 0.022 |
|  | JM-2L-DI | 0.002 | 1.602 | 0.037 | 0.000 | 0.714 | 0.016 | 0.001 | 0.080 | 0.022 |
|  | FCS-2L-DI | 0.002 | 1.604 | 0.037 | 0.000 | 0.711 | 0.016 | 0.001 | 0.079 | 0.022 |
|  | FCS-3L-ml.lmer | 0.002 | 1.553 | 0.037 | 0.000 | -0.468 | 0.016 | 0.001 | 0.118 | 0.022 |
|  | FCS-3L-Blimp | 0.002 | 1.570 | 0.037 | 0.000 | 0.683 | 0.016 | 0.001 | 0.083 | 0.022 |
| Low-high | ACA | 0.000 | -0.598 | 0.017 | -0.006 | -1.262 | 0.031 | -0.002 | -0.470 | 0.018 |
|  | JM-1L-DI-wide | 0.001 | 1.709 | 0.017 | 0.000 | 0.021 | 0.027 | 0.000 | 0.100 | 0.014 |
|  | FCS-1L-DI-wide | 0.001 | 1.696 | 0.017 | 0.000 | 0.018 | 0.027 | 0.001 | 0.103 | 0.014 |
|  | JM-2L-wide | 0.001 | 1.654 | 0.017 | 0.000 | 0.026 | 0.027 | 0.000 | 0.095 | 0.014 |
|  | FCS-2L-wide | 0.001 | 1.660 | 0.017 | 0.000 | 0.020 | 0.027 | 0.000 | 0.096 | 0.014 |
|  | JM-2L-DI | 0.001 | 1.767 | 0.017 | 0.000 | 0.066 | 0.027 | 0.001 | 0.111 | 0.014 |
|  | FCS-2L-DI | 0.001 | 1.763 | 0.017 | 0.000 | 0.066 | 0.027 | 0.001 | 0.108 | 0.014 |
|  | FCS-3L-ml.lmer | 0.001 | 1.674 | 0.017 | 0.000 | -0.025 | 0.027 | 0.001 | 0.124 | 0.014 |
|  | FCS-3L-Blimp | 0.001 | 1.728 | 0.017 | 0.000 | 0.063 | 0.027 | 0.001 | 0.112 | 0.014 |
| Low-low | ACA | 0.000 | -0.580 | 0.016 | 0.000 | -0.226 | 0.025 | -0.007 | -0.891 | 0.029 |
|  | JM-1L-DI-wide | 0.001 | 1.825 | 0.015 | 0.000 | 0.007 | 0.020 | 0.001 | 0.104 | 0.022 |
|  | FCS-1L-DI-wide | 0.001 | 1.820 | 0.015 | 0.000 | 0.007 | 0.020 | 0.001 | 0.104 | 0.022 |
|  | JM-2L-wide | 0.001 | 1.747 | 0.015 | 0.000 | 0.038 | 0.020 | 0.001 | 0.101 | 0.022 |
|  | FCS-2L-wide | 0.001 | 1.756 | 0.015 | 0.000 | 0.017 | 0.020 | 0.001 | 0.099 | 0.022 |
|  | JM-2L-DI | 0.001 | 1.865 | 0.015 | 0.000 | 0.275 | 0.020 | 0.001 | 0.086 | 0.022 |
|  | FCS-2L-DI | 0.001 | 1.872 | 0.015 | 0.000 | 0.274 | 0.020 | 0.001 | 0.084 | 0.022 |
|  | FCS-3L-ml.lmer | 0.001 | 1.797 | 0.015 | 0.000 | -0.115 | 0.020 | 0.001 | 0.118 | 0.022 |
|  | FCS-3L-Blimp | 0.001 | 1.817 | 0.015 | 0.000 | 0.267 | 0.020 | 0.001 | 0.084 | 0.022 |

^a^ Estimated bias (The average of the difference between the estimate and true value across the 1000 simulated datasets) ^b^ Empirical standard error from the 1000 estimated regression coefficients ^c^Average of the model based standard error across the 1000 replications

The following abbreviations are used to denote different MI methods, e.g., DI: dummy indicators, FCS: fully conditional specification,wide: repeated measures imputed in wide format,JM: joint modelling.

Table S13: The estimated regression coefficients (and standard errors) for the adjusting covariates, from available case analysis (ACA) and 8 MI approaches applied to the CATS data analysis.

| **Method** | **Wave*** | | **Age at wave 1 (years)*** | | **Female sex** | | **Standardized NAPLAN numeracy score at wave 1*** | | **SES at wave 1+** | | | | | | | | |
| --- | --- | --- | --- | --- | --- | --- | --- | --- | --- | --- | --- | --- | --- | --- | --- | --- | --- |
|  |  |  |  |  |  |  |  |  | **Quintile 1** | **Quintile 2** | | **Quintile 3** | | **Quintile 4** | | **Quintile 5** | |
| ACA | -0.011 | (0.006) | -0.207 | (0.053) | 0.148 | (0.039) | 0.715 | (0.020) | ref | -0.027 | (0.109) | -0.104 | (0.092) | 0.011 | (0.088) | -0.026 | (0.088) |
| JM-1L-DI-wide | -0.018 | (0.006) | -0.260 | (0.052) | 0.123 | (0.039) | 0.715 | (0.020) | ref | -0.058 | (0.107) | -0.106 | (0.092) | -0.006 | (0.089) | -0.043 | (0.086) |
| FCS-1L-DI-wide | -0.018 | (0.006) | -0.261 | (0.052) | 0.123 | (0.039) | 0.713 | (0.020) | ref | -0.063 | (0.107) | -0.115 | (0.089) | -0.007 | (0.085) | -0.037 | (0.086) |
| JM-2L-wide | -0.018 | (0.006) | -0.276 | (0.050) | 0.125 | (0.037) | 0.716 | (0.021) | ref | -0.069 | (0.111) | -0.093 | (0.097) | -0.011 | (0.088) | -0.046 | (0.087) |
| FCS-2L-wide | -0.019 | (0.006) | -0.253 | (0.052) | 0.121 | (0.039) | 0.715 | (0.020) | ref | -0.067 | (0.106) | -0.113 | (0.090) | -0.006 | (0.085) | -0.040 | (0.085) |
| JM-2L-DI | -0.017 | (0.006) | -0.239 | (0.052) | 0.121 | (0.038) | 0.715 | (0.020) | ref | -0.044 | (0.107) | -0.082 | (0.088) | 0.015 | (0.084) | -0.021 | (0.085) |
| FCS-2L-DI | - | - | - | - | - | - | - | - | - | - | - | - | - | - | - | - | - |
| FCS-3L-ml.lmer | -0.017 | (0.006) | -0.236 | (0.054) | 0.127 | (0.038) | 0.715 | (0.020) | ref | -0.031 | (0.106) | -0.085 | (0.090) | 0.022 | (0.089) | -0.021 | (0.086) |
| FCS-3L-Blimp | -0.017 | (0.006) | -0.233 | (0.051) | 0.128 | (0.037) | 0.715 | (0.020) | ref | -0.053 | (0.105) | -0.088 | (0.088) | 0.008 | (0.084) | -0.034 | (0.084) |

* Change in mean outcome (standardised NAPLAN numeracy score) per unit change in the predictor conditional on all other predictors in the model.

+ Change in mean outcome (standardised NAPLAN numeracy score) in the given quintile compared with quintile 1 conditional on all other predictors in the model.

SES = socio-economic status


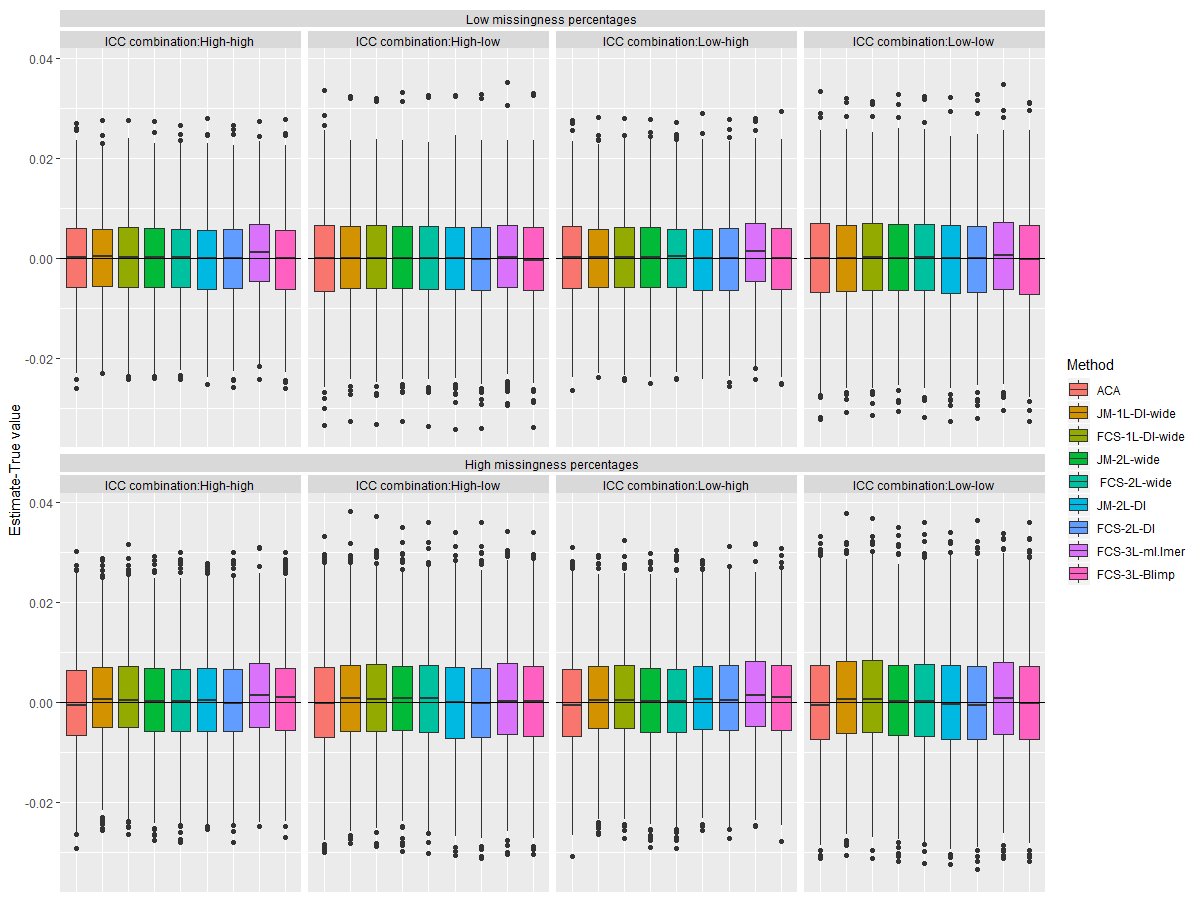


Figure S1: Distribution of the bias in the estimated regression coefficient of interest ($\beta_{1}, true value=-0.025)$ across the 1000 replications for available case analysis (ACA) and the 8 multiple imputation (MI) approaches under two scenarios for missing data proportions at waves 2, 4 and 6 (10%, 15%, 20% and 20%, 30%, 40%, respectively) and four ICC combinations when data are missing completely at random (MCAR)

The lower and upper margins of the boxes represent the 25^th^ (Q_1_) and the 75^th^ (Q_3_) percentiles of the distribution respectively. The whiskers extend to Q_1_-1.5*(Q_3_- Q_1_) at the bottom and Q_3_ +1.5*(Q_3_- Q_1_) at the top.

The following abbreviations are used to denote different MI methods, e.g., DI: dummy indicators, FCS: fully conditional specification, wide: repeated measures imputed in wide format,JM: joint modelling.


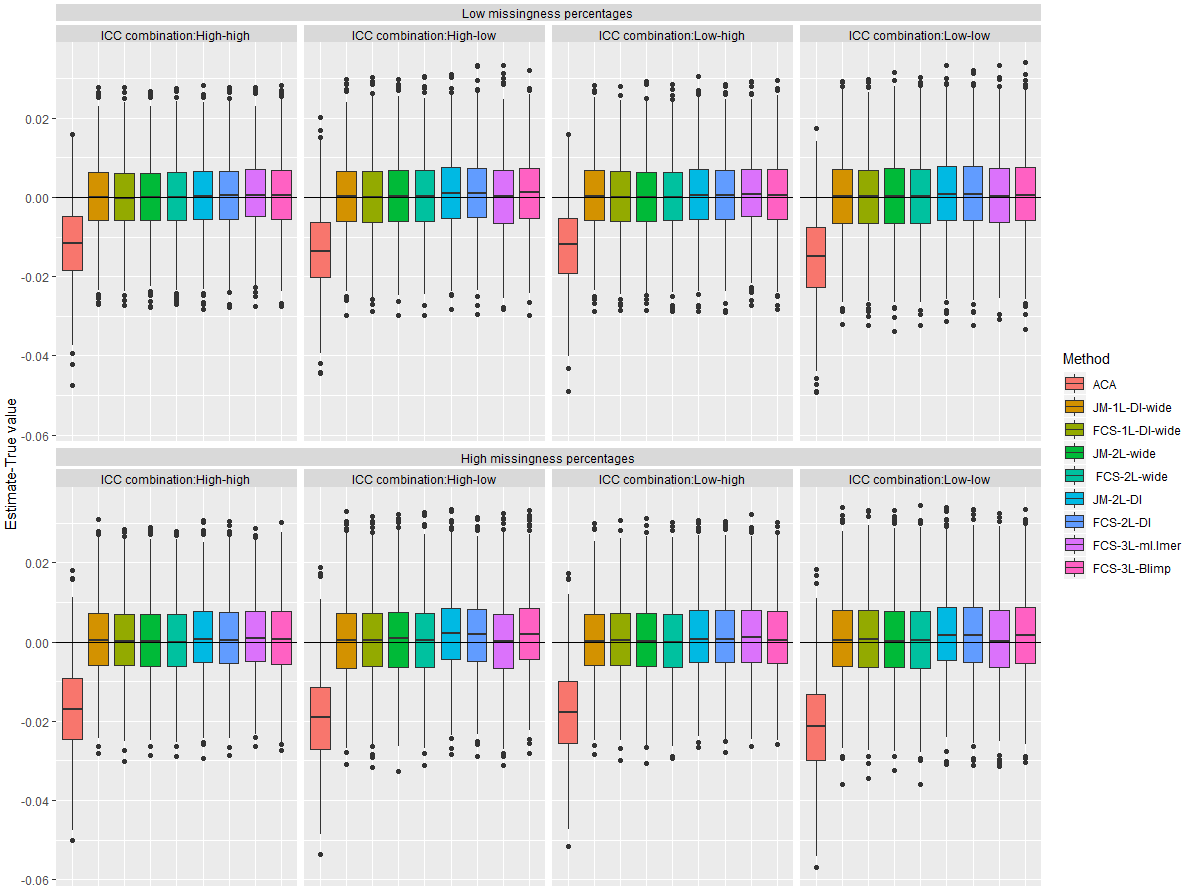


Figure S2: Distribution of the bias in the estimated regression coefficient of interest ($\beta_{1}, true value=-0.025)$ across 1000 replications for available case analysis (ACA) and the 8 multiple imputation (MI) approaches under two scenarios for missing data proportions at waves 2, 4 and 6 (10%, 15%, 20% and 20%, 30%, 40%, respectively) and four ICC combinations when data are missing at random (MAR-inflated)

The lower and upper margins of the boxes represent the 25^th^ (Q_1_) and the 75^th^ (Q_3_) percentiles of the distribution respectively. The whiskers extend to Q_1_-1.5*(Q_3_- Q_1_) at the bottom and Q_3_ +1.5*(Q_3_- Q_1_) at the top.

The following abbreviations are used to denote different MI methods, e.g., DI: dummy indicators, FCS: fully conditional specification, wide: repeated measures imputed in wide format, JM: joint modelling.


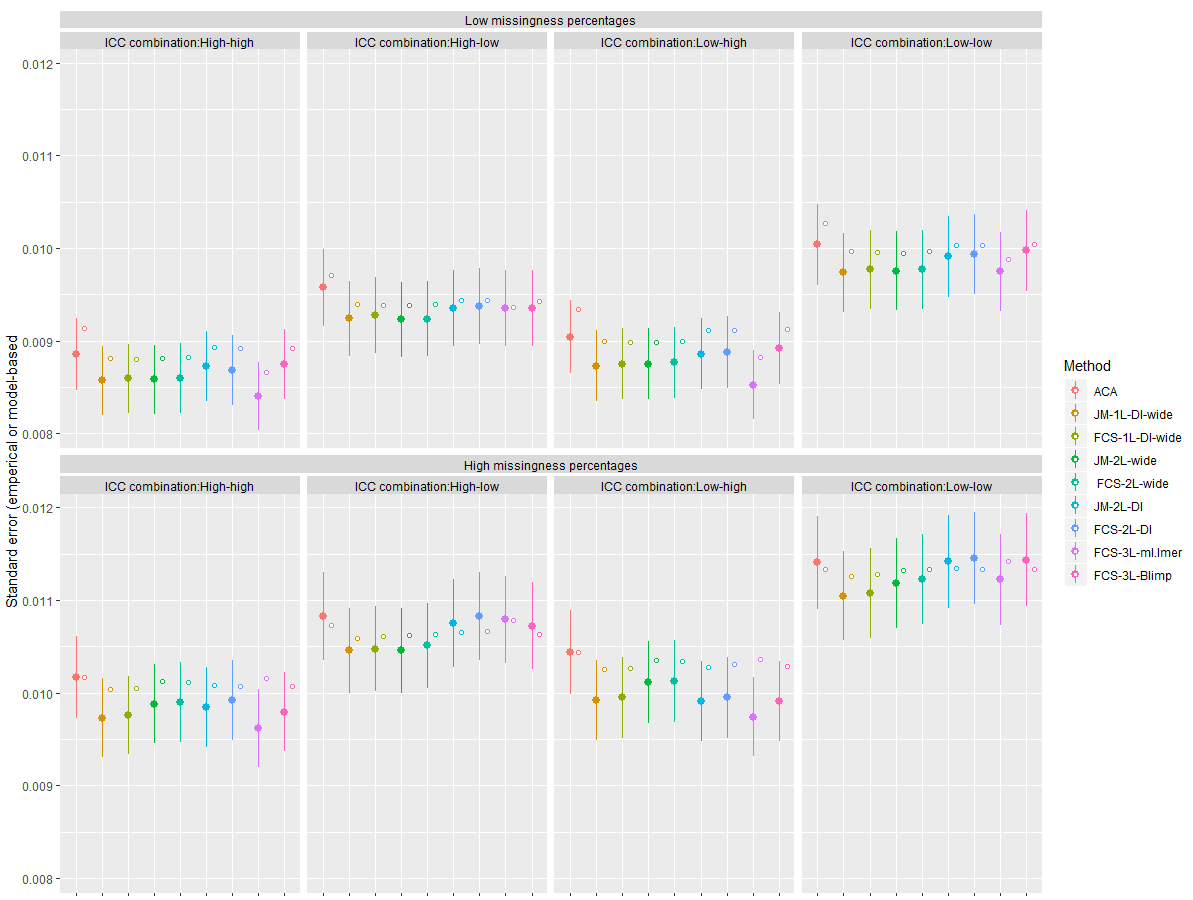


Figure S3: Empirical standard errors (filled circles with error bars showing ±1.96× Monte Carlo standard errors) and average model-based standard errors (hollow circles) from 1000 replications, for available case analysis (ACA) and the 8 multiple imputation (MI) approaches under two scenarios for missing data proportions at waves 2,4 and 6 (10%, 15%, 20% and 20%, 30%, 40%, respectively)and four ICC combinations when data are missing completely at random (MCAR)

The following abbreviations are used to denote different MI methods, e.g., DI: dummy indicators, FCS: fully conditional specification, wide: repeated measures imputed in wide format, JM: joint modelling.


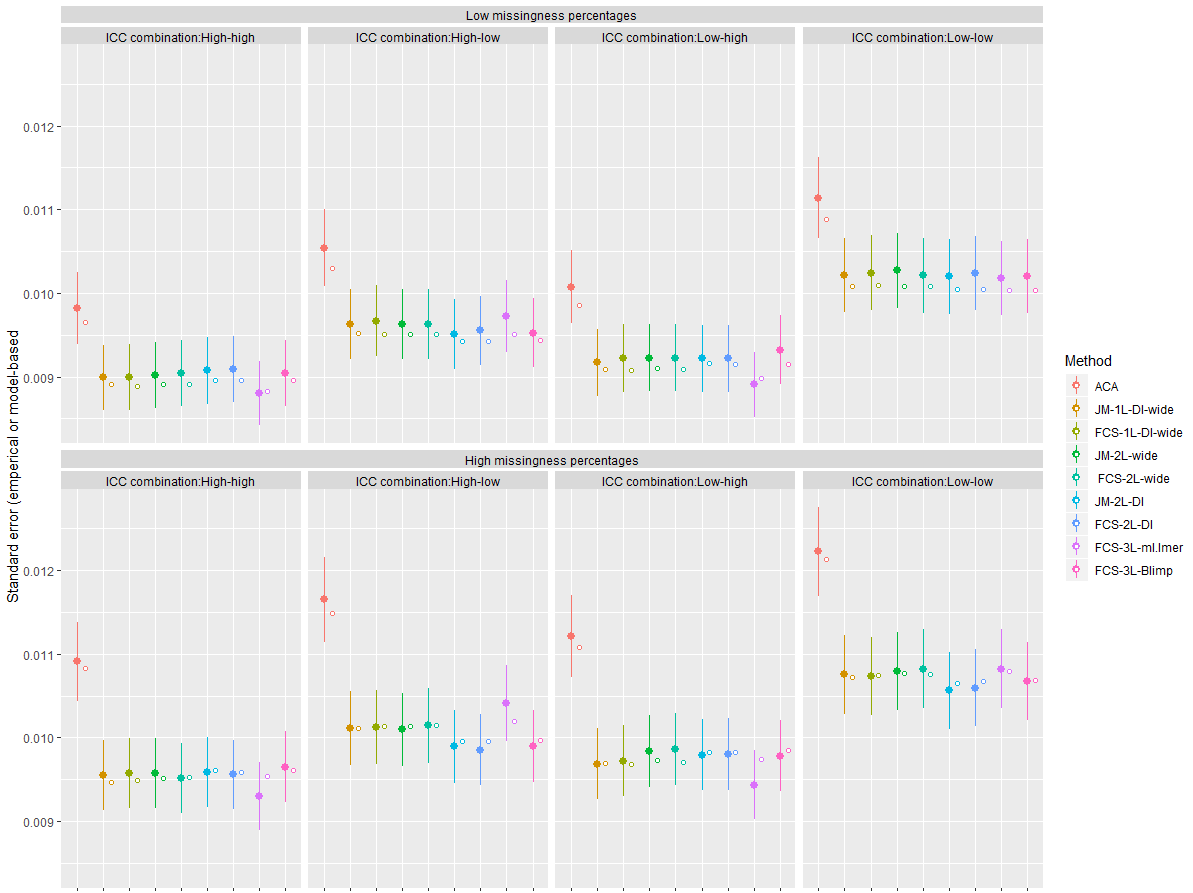


Figure S4: Empirical standard errors (filled circles with error bars showing ±1.96× Monte Carlo standard errors) and average model-based standard errors (hollow circles) from 1000 replications, for available case analysis (ACA) and the 8 multiple imputation (MI) approaches under two scenarios for missing data proportions at waves 2,4 and 6 (10%, 15%, 20% and 20%, 30%, 40%, respectively)and four ICC combinations when data are missing at random (MAR-inflated)

The following abbreviations are used to denote different MI methods, e.g., DI: dummy indicators, FCS: fully conditional specification, wide: repeated measures imputed in wide format, JM: joint modelling.


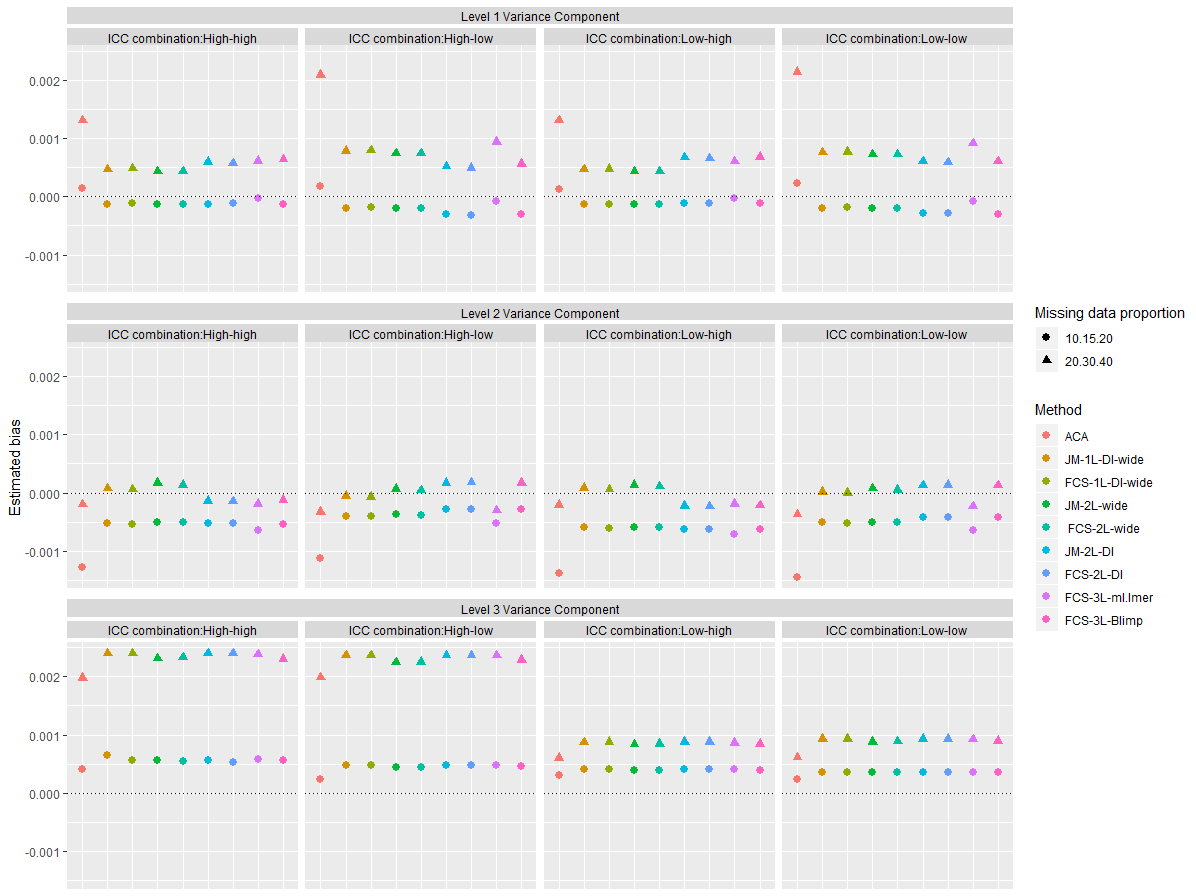

Figure S5: Estimated bias in the variance components at level 1, 2 and 3 across the 1000 simulated datasets available case analysis (ACA) and the 8 multiple imputation (MI) approaches under two scenarios for missing data proportions at waves 2, 4 and 6 (10%, 15%, 20% and 20%, 30%, 40%, respectively) and four ICC combinations when data are missing completely at random (MCAR).

The following abbreviations are used to denote different MI methods, e.g., DI: dummy indicators, FCS: fully conditional specification, wide: repeated measures imputed in wide format, JM: joint modelling.


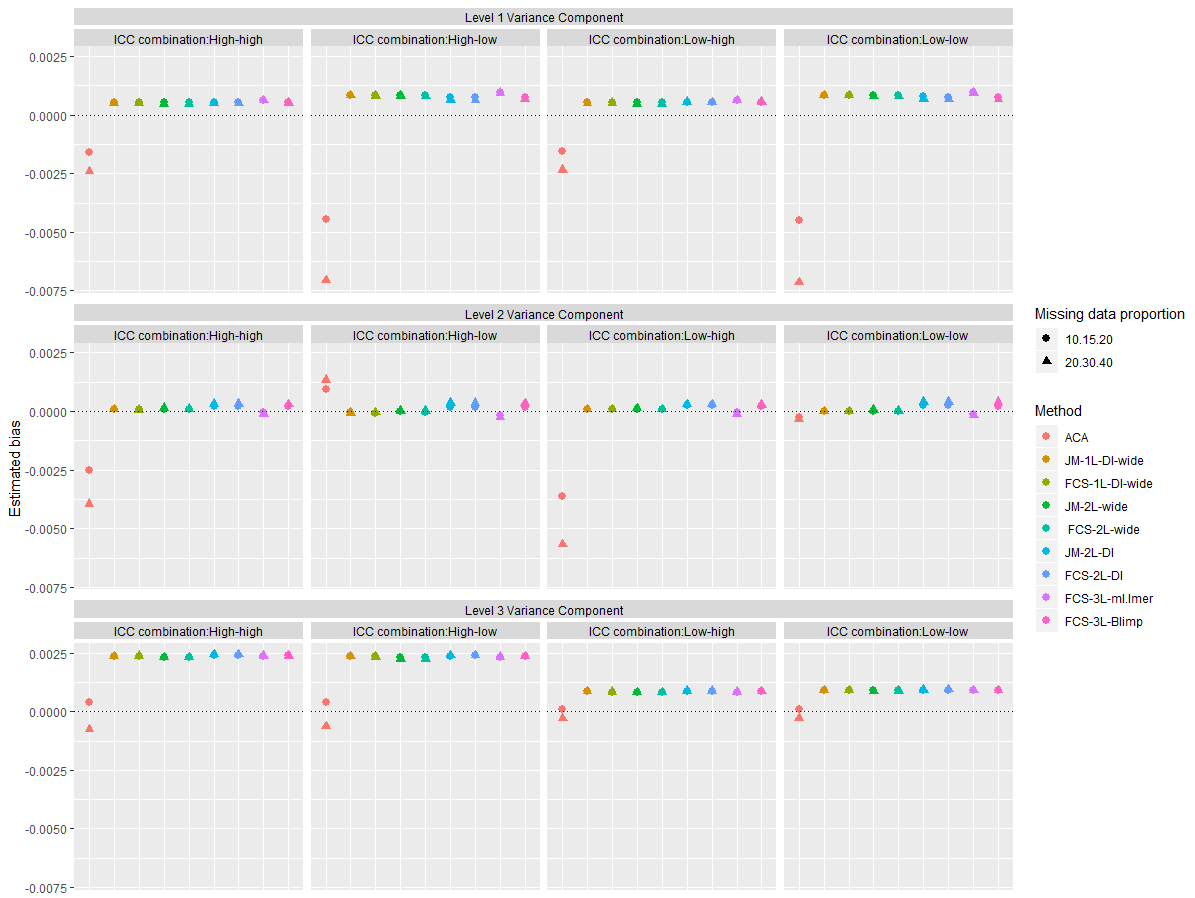


Figure S6: Estimated bias in the variance components at level 1, 2 and 3 across the 1000 simulated datasets available case analysis (ACA) and the 8 multiple imputation (MI) approaches under two scenarios for missing data proportions at waves 2, 4 and 6 (10%, 15%, 20% and 20%, 30%, 40%, respectively) and four ICC combinations when data are missing at random (MAR-inflated).

The following abbreviations are used to denote different MI methods, e.g., DI: dummy indicators, FCS: fully conditional specification, wide: repeated measures imputed in wide format, JM: joint modelling.
