# Supplementary material for: Evaluation of approaches for multiple imputation of three-level data
Source: BMC Med Res Methodol. 2020 Aug 12;20:207. doi: 10.1186/s12874-020-01079-8 (PMC7422505; doi:10.1186/s12874-020-01079-8)

**Illustration of the MI approaches for handling incomplete three-level data**

Here, we provide the R syntax for the MI approaches as applied to the real CATS data. A detailed description of the variables and the data are provided in the main text.

The following packages will be required


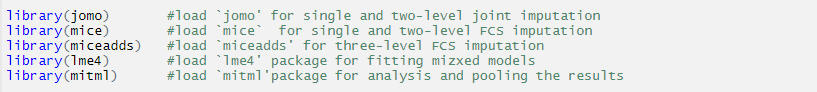


The variables used in the imputation models are as follows:


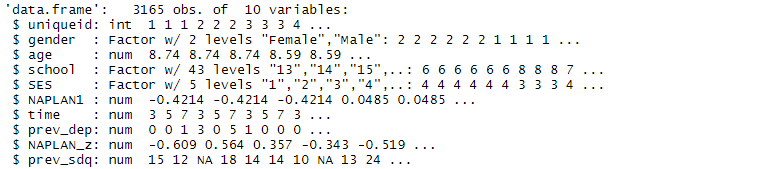


The time varying variables prev_dep (depressive symptoms at the previous wave), NAPLAN_z (NAPLAN numeracy scores) and prev_sdq (overall child behaviour reported by SDQ) all have missing values, while all the other variables are completely observed.

**Single level JM with DI for schools with repeated measures analysed in wide format (JM-1L-DI-wide)**

The original data above is in long format, with one row per individual per wave. For JM-1L-DI-wide, the data will need to be reshaped into wide format: one row per individual, with the repeated measures (at waves 3, 5 and 7) of the time varying variables as distinct variables. For this the reshape function can be used.


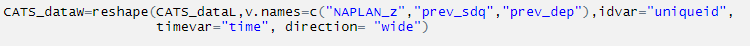


In order to guarantee that the correct function is being used by `jomo’, make sure that continuous variables are stored as numeric vectors in the data frame and binary/categorical variables as factors. To include fully observed categorical covariates with three or more categories in the predictor matrix (X) of the imputation model, appropriate dummy variables will have to be created.

Under this approach, the incomplete variables prev_dep.3, prev_dep.5, prev_dep.7, NAPLAN_z.3, NAPLAN_z.5, NAPLAN_z.7, prev_sdq.3, prev_sdq.5 and prev_sdq.7 will need to be specified as the outcomes(Y) in the single level imputation model, while all complete variables will serve as the predictors(X). The imputation model can be specified as follows:


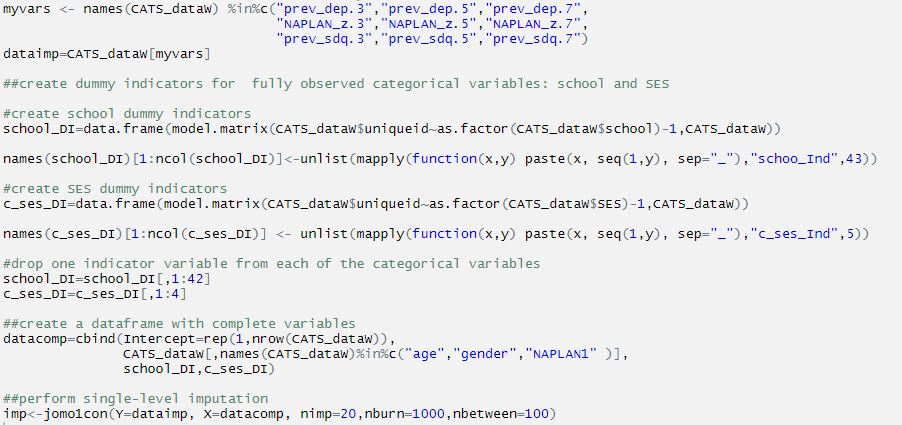


After imputation, the imputed datasets will need to be reshaped into long format before fitting the substantive analysis model.
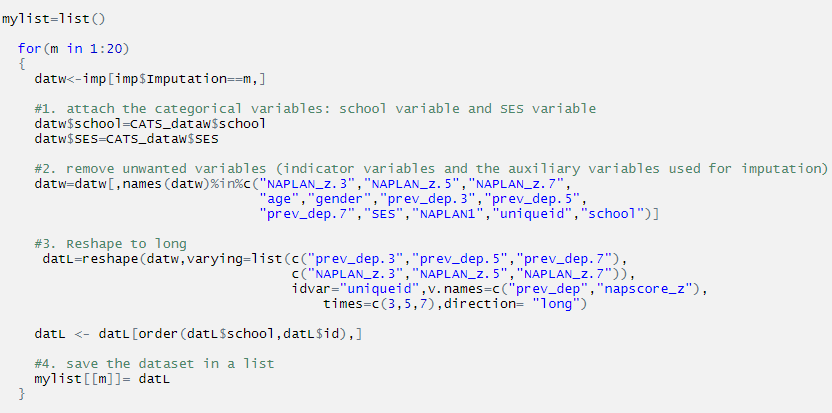


For fitting the analysis model on the imputed datasets and pooling the results, the `mitml’ package can be used.


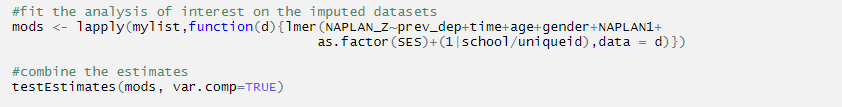


**Single level FCS with DI for schools with repeated measures analysed in wide format (FCS-1L-DI-wide)**

Similar to the JM-1L-DI-wide approach, here the dataset will need to be in wide format during the imputation process. To carry out single-level FCS, R package `mice’ can be used. Because all the incomplete variables prev_dep.3, prev_dep.5, prev_dep.7, NAPLAN_z.3, NAPLAN_z.5, NAPLAN_z.7, prev_sdq.3, prev_sdq.5 and prev_sdq.7 are continuous, the method used is `norm`. Here each of the incomplete variable will be imputed by using a series of univariate imputation models with the incomplete variable as the outcome and all the other variables as the predictors. Because we don’t need the individual’s id, `uniqueid’ to serve as a predictor in the imputation model it is removed from the dataset.


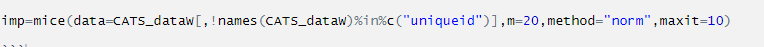


After carrying out MI, as before the imputed datasets will need to be reshaped back to long format prior to fitting the substantive analysis model.

**Two level JM for school clusters with repeated measures analysed in wide format (JM-2L-wide)**

Under this approach, the repeated measures will again need to be reshaped into wide format during the imputation stage and then the two-level joint imputation model in `jomo’ can be applied for the school clusters.

The incomplete variables, prev_dep.3, prev_dep.5, prev_dep.7, NAPLAN_z.3, NAPLAN_z.5, NAPLAN_z.7, prev_sdq.3, prev_sdq.5 and prev_sdq.7, will be specified as outcomes(Y) in the two level imputation model, while all complete variables will serve as the predictors(X). As before to include fully observed categorical covariates with three or more categories in the predictor matrix of the imputation model, appropriate dummy variables have to be created. In this case because the clustering due to schools will be modelled using random effects (random intercepts) and not as a fixed effect as in SL-JM-DI-wide, dummy variables will need to be created only for SES.


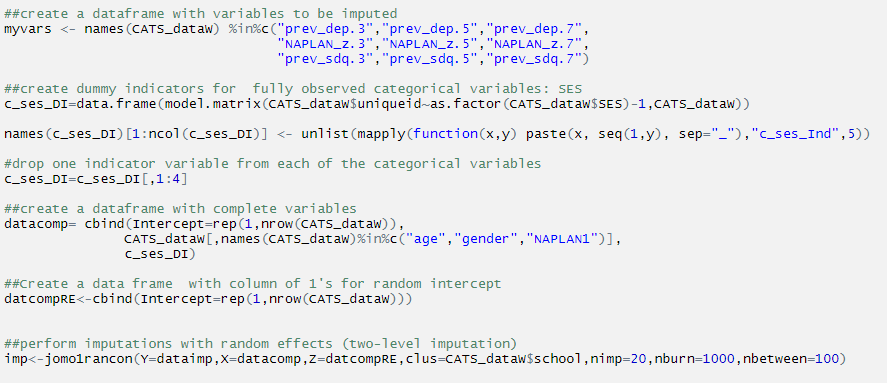


However here, an additional data frame (Z) will also need to be created for the random intercepts of the school clusters and the school cluster indicator will need to be specified in the imputation model as shown above.

After imputation, the imputed datasets will need to be reshaped into long format before fitting the substantive analysis model.


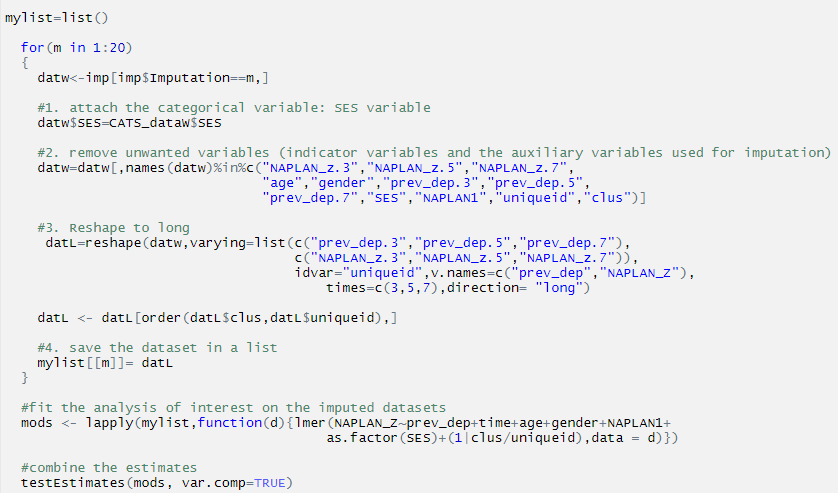


**Two level FCS for school clusters with repeated measures analysed in wide format (FCS-2L-wide)**

Similar to JM-2L-wide, under this approach the repeated measures will need to be reshaped into wide format during the imputation stage .Then two-level univariate imputation models are specified for each incomplete repeated measure. To specify the univariate models R package `mice’ can be used. Here for each univariate imputation model an imputation method and the predictor variables to be used will need to be specified. Below for each incomplete variable, prev_dep.3, prev_dep.5, prev_dep.7, NAPLAN_z.3, NAPLAN_z.5, NAPLAN_z.7, prev_sdq.3, prev_sdq.5 and prev_sdq.7, the imputation method to be used is specified as `2l.pan’


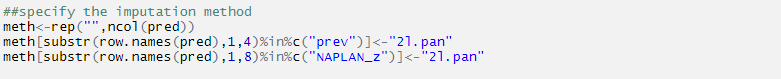


Then the predictor matrix can be specified as shown below. Each row will indicate the variable being imputed (the outcome in the univariate imputation model) while the columns corresponds the predictors with the integer values denoting the relations between the outcome and predictor variables.


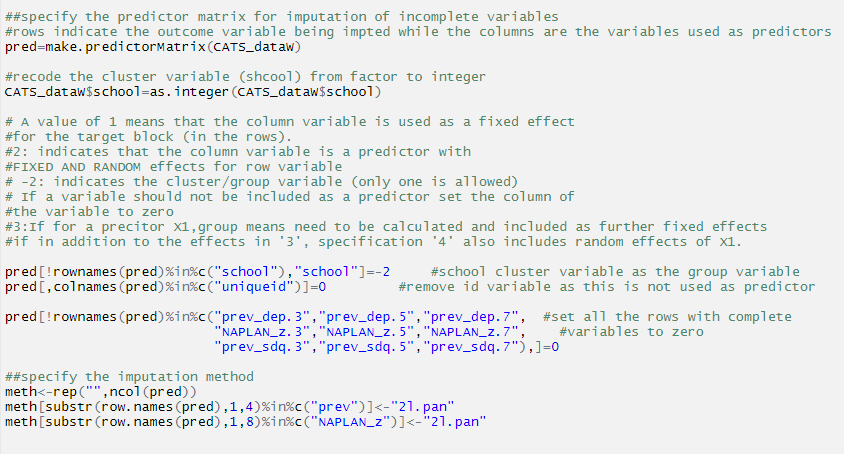


The imputation can be then carried out as follows


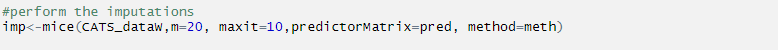


After imputation, the imputed datasets will need to be reshaped into long format before fitting the substantive analysis model as before.

**Two level JM for repeated measures with DIs for the school clusters (JM-2L-DI)**

Under this approach, the data can be imputed in long format because the clustering between repeated measures of the same variable is now modelled using the two-level joint imputation model in `jomo’.

The incomplete variables, prev_dep, NAPLAN_z and prev_sdq , will be specified as outcomes(Y) in the two level imputation model, while all complete variables will serve as the predictors(X). As before to include fully observed categorical covariates with three or more categories in the predictor matrix of the imputation model, appropriate dummy variables have to be created. An additional data frame (Z) will also need to be created for the random intercepts for each individual and the cluster indicator, individual’s uniqueid, will need to be specified in the imputation model as shown below.


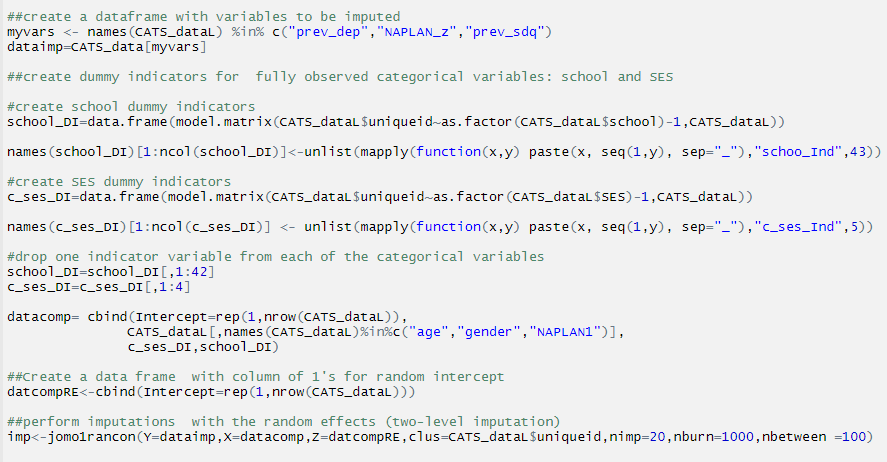


**Two level FCS for repeated measures with DIs for the school clusters (FCS-2L-DI)**

Similar to JM-2L-DI here the repeated measures will be imputed in long format, with the clustering between repeated measures of the same variable modelled using a univariate two-level imputation model. The imputation models can be specified using the R package `mice’. For each univariate imputation model, an imputation method and the predictor variables to be used will need to be specified as shown below. In this case we will have to specify three univariate imputation models each for incomplete variable prev_dep, NAPLAN_z and prev_sdq.


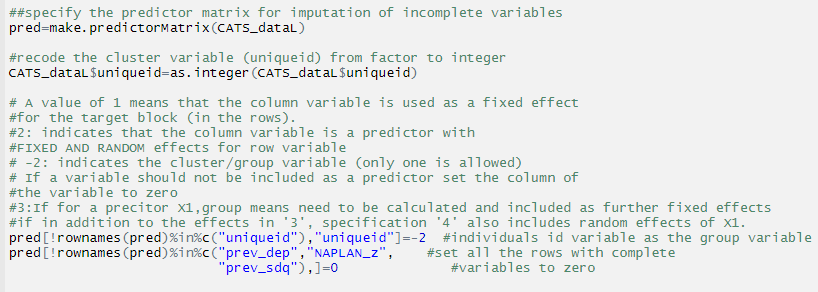


Then specify the imputation method and perform imputations


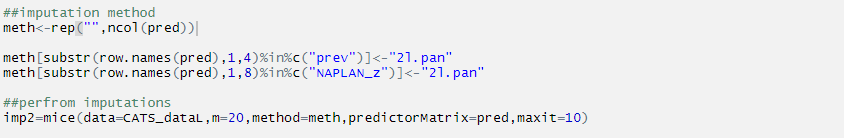


**Three level FCS in ml.lmer (FCS-3L)**

Under this approach a three-level imputation model can be specified for the imputation of the missing values in the time varying variables, prev_dep, NAPLAN_z and prev_sdq, using the R package `miceadds’. As a three-level model is used, the repeated measures will be imputed in long format.

As in the previous FCS approaches, a predictor matrix and an imputation method will need to be specified for each incomplete variable.


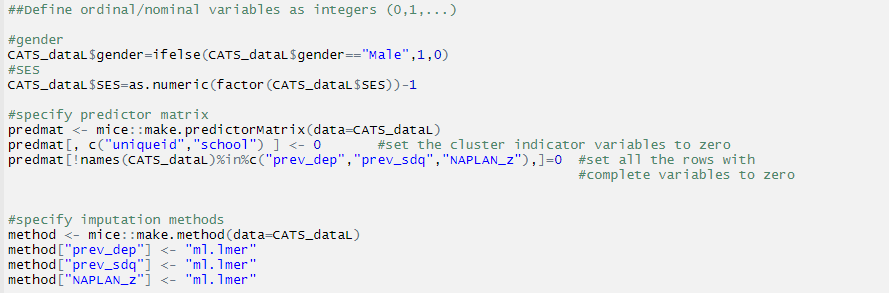


In addition to the predictor matrix, for variables that will be imputed using the method ml.lmer, the hierarchical structure must be set with two additional arguments (i.e. outside the predictor matrix). For all higher-level variables (i.e. those at level 2 and level 3) we need to specify the level at which the variables are measured. For rest of the variables (i.e. for level 1 variables) the level can be left blank.


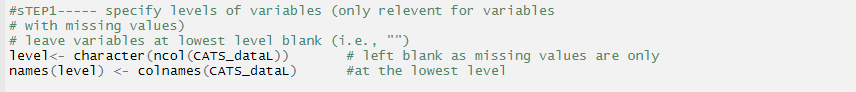


Next for each variable being imputed, cluster variables that define the hierarchical structure in the imputation model must be specified as follows. By default, this uses a random intercept model with random effects at each of the specified levels.


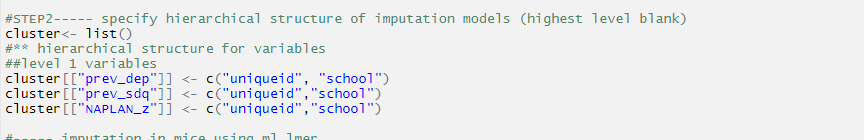

Then the imputation model can be specified as follows:


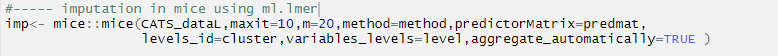


**Three level FCS in Blimp (FCS-3L)**

Note: The syntax shown below is of the Blimp Version 1.1 used in this paper (which is an older version of the current operational version). The newest version of the Blimp software with the user guide and examples with the current syntax can be found at [www.appliedmissingdata.com/multilevel-imputation](https://protect-au.mimecast.com/s/MznVCoV1VwsrWyAmczopgA?domain=appliedmissingdata.com)

Similar to the ml.lmer approach, this approach will also use a three-level imputation model and therefore the repeated measures will be imputed in long format.


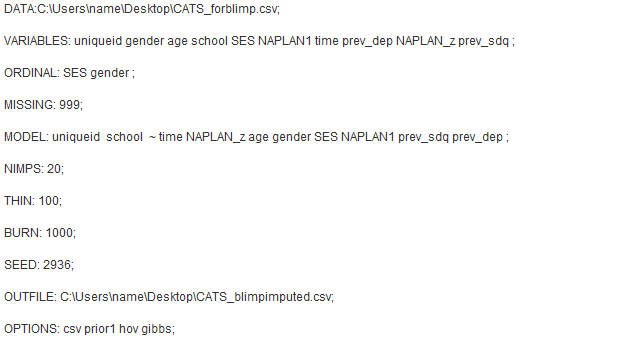

Supplement: Supplementary file 2 — Additional file 2. R syntax for the CATS data illustration [file 12874_2020_1079_MOESM2_ESM.docx]
